# Tissue-Specific Orchestration of Gilthead Sea Bream Resilience to Hypoxia and High Stocking Density

**DOI:** 10.3389/fphys.2019.00840

**Published:** 2019-07-10

**Authors:** Juan Antonio Martos-Sitcha, Paula Simó-Mirabet, Verónica de las Heras, Josep Àlvar Calduch-Giner, Jaume Pérez-Sánchez

**Affiliations:** Nutrigenomics and Fish Growth Endocrinology, Institute of Aquaculture Torre de la Sal (IATS-CSIC), Castellón, Spain

**Keywords:** hematology, hypometabolism, hypoxia, limiting oxygen saturation, *Sparus aurata*, stocking density, tissue-specific transcriptomics

## Abstract

Two different O_2_ levels (normoxia: 75–85% O_2_ saturation; moderate hypoxia: 42–43% O_2_ saturation) and stocking densities (LD: 9.5, and HD: 19 kg/m^3^) were assessed on gilthead sea bream (*Sparus aurata*) in a 3-week feeding trial. Reduced O_2_ availability had a negative impact on feed intake and growth rates, which was exacerbated by HD despite of the improvement in feed efficiency. Blood physiological hallmarks disclosed the enhancement in O_2_-carrying capacity in fish maintained under moderate hypoxia. This feature was related to a hypo-metabolic state to cope with a chronic and widespread environmental O_2_ reduction, which was accompanied by a differential regulation of circulating cortisol and growth hormone levels. Customized PCR-arrays were used for the simultaneous gene expression profiling of 34–44 selected stress and metabolic markers in liver, white skeletal muscle, heart, and blood cells. The number of differentially expressed genes ranged between 22 and 19 in liver, heart, and white skeletal muscle to 5 in total blood cells. Partial Least-Squares Discriminant Analysis (PLS-DA) explained [R2Y(cum)] and predicted [Q2Y(cum)] up to 95 and 65% of total variance, respectively. The first component (R2Y = 0.2889) gathered fish on the basis of O_2_ availability, and liver and cardiac genes on the category of energy sensing and oxidative metabolism (*cs, hif-1*α, *pgc1*α, *pgc1*β, *sirt*s *1*-*2*-*4*-*5*-*6*-*7*), antioxidant defense and tissue repair (*prdx5, sod2, mortalin, gpx4, gr, grp-170*, and *prdx3*) and oxidative phosphorylation (*nd2, nd5*, and *coxi*) highly contributed to this separation. The second component (R2Y = 0.2927) differentiated normoxic fish at different stocking densities, and the white muscle clearly promoted this separation by a high over-representation of genes related to GH/IGF system (*ghr-i, igfbp6b, igfbp5b, insr, igfbp3*, and *igf-i*). The third component (R2Y = 0.2542) discriminated the effect of stocking density in fish exposed to moderate hypoxia by means of hepatic fatty acid desaturases (*fads2, scd1a*, and *scd1b)* and muscle markers of fatty acid oxidation (*cpt1a*). All these findings disclose the different contribution of analyzed tissues (liver ≥ heart > muscle > blood) and specific genes to the hypoxic- and crowding stress-mediated responses. This study will contribute to better explain and understand the different stress resilience of farmed fish across individuals and species.

## Introduction

Several attempts have been made over the course of last years to monitor the ecological and physiological impacts of a reduced O_2_ availability in aquatic environments (Ekau et al., 2010; Richards, 2011; Zhu et al., 2013; Deutsch et al., 2015). The magnitude and orchestration of adaptive responses from a physiological point of view, including blood hematology and metabolic regulation, reflects the duration and intensity of hypoxic stimuli in different marine species (Martos-Sitcha et al., 2017; Cadiz et al., 2018), being defined the limiting O_2_ saturation (LOS) as the threshold level where regulatory mechanisms are no longer sufficient to maintain O_2_ consumption without compromising any physiological function (Remen et al., 2015, 2016). To minimize hypoxia impact, fish reduce feed intake and reorganize its metabolism to limit the tissue O_2_ demand (Hopkins and Powell, 2001; Bermejo-Nogales et al., 2014b). This allows to preserve aerobic metabolism by means of a restricted mitochondrial respiration and a shift in substrate preferences, as it has been reported in humans and rodents during the metabolic adaption of skeletal muscle to high altitude hypoxia (Murray, 2009). Other adaptive responses include changes in the production and scavenging of reactive oxygen species (ROS) (Lushchak and Bagnyukova, 2006; Bermejo-Nogales et al., 2014a), gill surface functionality (Nilsson, 2007) and hemoglobin (Hb)-O_2_ binding characteristics (Jensen and Weber, 1982; Nikinmaa, 2001). In most aquaculture scenarios, these adaptive features are commonly associated to increases in temperature and high stocking rearing densities (Person-Le Ruyet et al., 2008; Vikeså et al., 2017), which in turn can compromise water quality resulting in impaired fish growth and immunity (Pickering, 1993; Van Weerd and Komen, 1998; Montero et al., 1999; Ashley, 2007). Indeed, Arctic charr (*Salvelinus alpinus*) or meager (*Argyrosomus regius*) kept at high stocking densities evidenced a better growth performance than fish reared at low densities as long as water quality was preserved (Jørgensen et al., 1993; Millán-Cubillo et al., 2016). The impact of crowding stress can also be minimized when O_2_ levels are not below LOS (Ruer et al., 1987; Araújo-Luna et al., 2018). Unraveling the combined effects of hypoxia and high rearing density are, thereby, crucial to warrant welfare during intensive fish farming.

Progress toward a more sustainable and environmentally friendly aquaculture requires important investments in both conventional and new methodologies for a less invasive and more refined phenotyping of individual farmed fish. Main achievements so far include the use of acoustic telemetry or stand-alone biosensors for the non-disturbing monitoring of feeding behavior or metabolic capabilities (Føre et al., 2017; Martos-Sitcha et al., 2019). In addition to that, major progress has been done with the advent of wide-holistic omics based on functional genomics, proteomics, metabolomics and metagenomics as powerful toolsets for the development of a highly technified aquaculture in different fish species (Yáñez et al., 2015; Martin and Król, 2017; Martínez-Porchas and Vargas-Albores, 2017; Alfaro and Young, 2018; Rodrigues et al., 2018). Such approaches are increasingly used in gilthead sea bream (*Sparus aurata*), a highly and economically important cultured fish species in the Mediterranean area. Thus, a first draft genome based on genetic-linkage maps (Pauletto et al., 2018) and other current genome initiatives will contribute to have major progress in selective breeding and epigenetic research in gilthead sea bream. Also, in this species, important research efforts have been conducted to define a reference pattern for skin/intestine mucus proteome (Estensoro et al., 2016; Pérez-Sánchez et al., 2017), gut microbiota (Piazzon et al., 2017), or serum metabolome (Gil-Solsona et al., 2019). Moreover, the use of high-density microarrays (Calduch-Giner et al., 2010, 2012, 2014), pathway-focused PCR-arrays (Benedito-Palos et al., 2014, 2016; Bermejo-Nogales et al., 2014b, 2015; Pérez-Sánchez et al., 2015; Magnoni et al., 2017; Martos-Sitcha et al., 2017) and more recently NGS (Piazzon et al., 2019) have contributed to define tissue-specific gene expression patterns in response to nutritional, environmental and parasite challenges. Likewise, the synchronization of the molecular clock of sea bream larvae, involving more than 2,500 genes with a clear circadian rhythmicity, has been proposed as certification of juvenile quality later in life (Yúfera et al., 2017). In the present study, we aim to go further on the definition of criteria of fish welfare and quality, regarding in depth the effect of two different initial stocking densities (9.5 kg/m^3^, 19 kg/m^3^) and O_2_ saturation levels (85%, 42–43% O_2_ saturation) in a 3-week trial with fast growing juveniles of gilthead sea bream. The analyzed parameters included the gene expression pattern of a set of growth and metabolic markers of liver, skeletal muscle, heart and blood cells in combination with data on growth performance, as well as blood hematology and biochemistry. The working hypothesis is that each tissue contributes differentially to the homeostatic load achievement, helping the generated knowledge to better exploit the plasticity and resilience of gilthead sea bream under stressful conditions.

## Materials and Methods

### Animal Care

Gilthead sea bream juveniles of Atlantic origin (Ferme Marine du Douhet, Bordeaux, France) were reared from early life stages in the indoor experimental facilities of Institute of Aquaculture Torre de la Sal (IATS-CSIC, Spain) under natural photoperiod and temperature conditions (40°5^′^N; 0°10^′^E). Sea water was pumped ashore (open system) and filtered through a 10-μm filter. The O_2_ content of water effluents in standard conditions was always higher than 85% saturation, and unionized ammonia remained below 0.02 mg/L.

### Experimental Set-Up and Sampling

Juvenile fish (initial body weight 34.08 ± 0.31 g) were randomly distributed in 12 90 L tanks coupled to a recirculation system equipped with physical and biological filters, and programmable temperature and O_2_ devices (Supplementary Figure 1). Water temperature was daily monitored and maintained at 25-27°C. Fish were arbitrarily allocated to constitute two different initial stocking densities (six tanks per condition) fed once daily to visual satiety with a commercial diet (EFICO Forte 824, BioMar, Palencia, Spain): LD (low density, 25 fish/tank, 9.5 kg/m^3^) and HD (high density, 50 fish/tank, 19 kg/m^3^). Fish were allowed to acclimate to these conditions for 12 days before any manipulation. Fish behavior during acclimation was assessed by routine camera monitoring, and also by visual observation regarding to normal feeding performance. After the acclimation period, the water parameters of three tanks of each initial stocking density were kept unchanged, constituting the normoxic (>5.5 ppm O_2_; >85% O_2_ saturation) groups of each experimental condition (LDN, low density normoxia; HDN, high density normoxia). Fish maintained in the remaining six tanks experienced a gradual decrease in the water O_2_ level until reaching 3.0 ppm (42–43% O_2_ saturation), constituting the hypoxic groups of each experimental condition (LDH, low density hypoxia; HDH, high density hypoxia). The normal range of variation in O_2_ levels was marked by a rapid drop (3.8–4 ppm normoxic groups; 2.3 ppm hypoxic groups) 15–30 min after feeding, with a rapid restoration of reference values in less than 1 h by the automatic entrance of clean water from the main reservoir tank. This system allowed maintaining unionized ammonia below toxic levels (<0.50 mg/L) in both HDN and HDH groups.

After 22 days under these experimental conditions and following overnight fasting (>20 h after last meal), 12 fish (4 per tank) per experimental condition (LDN, LDH, HDN, and HDH) were anesthetized with 3-aminobenzoic acid ethyl ester (100 mg/L), weighed and blood was taken from caudal vessels with EDTA-treated syringes (less than 5 min for all the fish sampled for each tank). All lethal samples were collected between 10.00 am and 12.00 am to reduce the biologic variability due to circadian rhythms and postprandial-mediated effects, since temperatures used in the present experimental approach (25-27°C) ensured that digestion processes have been completed (Gómez-Requeni et al., 2003). One blood aliquot (25 μL) was directly collected into a microtube containing 500 μL of stabilizing lysis solution (REAL total RNA spin blood kit, Durviz, Valencia, Spain) and stored at -80°C until total RNA extraction. Other aliquots were processed for haematocrit (Ht), Hb, and red blood cells (RBC) counting. The remaining blood was centrifuged at 3,000 × *g* for 20 min at 4°C, and plasma samples were frozen and stored at -20°C until biochemical and hormonal analyses were performed. Prior to tissue collection, fish were killed by cervical section. Liver and viscera were weighed, and representative biopsies of liver, dorsal skeletal muscle, and complete hearts were immediately snap-frozen in liquid nitrogen and stored at -80°C until extraction of total RNA.

### Blood Biochemistry and Hormonal Parameters

Measures of Ht were conducted using heparinized capillary tubes centrifuged at 1,500 × *g* for 30 min in a Sigma 1-14 centrifuge (Sigma, Germany). Hb was assessed using a Hemocue Hb 201+ (Hemocue, Sweden). Counts of RBC were made in a Neubauer chamber, using an isotonic solution (1% NaCl). Plasma glucose was analyzed using the glucose oxidase method (Thermo Electron, Louisville, CO, United States). Lactate was measured in deproteinized samples (perchloric acid 8%) by an enzymatic method based on the use of lactate oxidase and peroxidase (SPINREACT S.A., Girona, Spain). Total antioxidant capacity in plasma samples was measured with a commercial kit (Cayman Chemical, Ann Arbor, MI, United States) adapted to 96-well microplates. This assay relies on the ability of antioxidants in the samples to inhibit the oxidation of ABTS [2,2^′^-azino-di-(3-ethylbenzthiazoline sulphonate)] to ABTS radical cation by metmyoglobin, a derivatized form of myoglobin. The capacity of the sample to prevent ABTS oxidation is compared with that of Trolox (water-soluble tocopherol analog) and is quantified as mM Trolox equivalents. Plasma cortisol levels were analyzed using an EIA kit (Kit RE52061m IBL, International GmbH, Germany). The limit of detection of the assay was 3.01 ng/mL with intra- and inter-assay coefficients of variation lower than 3 and 5%, respectively. Plasma growth hormone (Gh) was determined by a homologous gilthead sea bream RIA as reported elsewhere (Martínez-Barberá et al., 1995). The sensitivity and midrange (ED50) of the assay where 0.15 and 1.8 ng/mL, respectively. Plasma insulin-like growth factors (Igf) were extracted by acid–ethanol cryoprecipitation (Shimizu et al., 2000), and the concentration of Igf-I was measured by means of a generic fish Igf-I RIA validated for Mediterranean perciform fish (Vega-Rubín de Celis et al., 2004). The sensitivity and midrange of the assay were 0.05 and 0.7–0.8 ng/mL, respectively.

### Gene Expression Analysis

Total RNA from liver, white muscle and heart was extracted using a MagMax-96 total RNA isolation kit (Life Technologies, Carlsbad, CA, United States), whereas total RNA from total blood cells was extracted using the REAL total RNA spin blood kit including a DNase step. The RNA yield in all tissues was >3.5 μg, with absorbance measures (A_260/280_) of 1.9–2.1. Synthesis of cDNA was performed with the High-Capacity cDNA Archive Kit (Applied Biosystems, Foster City, CA, United States) using random decamers and 500 ng of total RNA in a final volume of 100 μL. Reverse transcription (RT) reactions were incubated 10 min at 25°C and 2 h at 37°C. Negative control reactions were run without RT.

The 96-well PCR-array layout was designed for the simultaneous profiling of a panel of 43 (liver), 44 (white muscle and total blood cells) or 34 (heart) genes, including markers of GH/IGF system (13), lipid metabolism (10), energy sensing and oxidative metabolism (12), antioxidant defense and tissue repair (10), muscle growth and cell differentiation (8), respiration uncoupling (3), xenobiotic metabolism (2), nuclear receptors (3), transmembrane translocation (8), mitochondrial dynamics and apoptosis (5), as well as OXPHOS (22) (Table 1). qPCR reactions were performed using an iCycler IQ Real-time Detection System (Bio-Rad, Hercules, CA, United States). Diluted RT reactions were conveniently used for qPCR assays in a 25 μL volume in combination with a SYBR Green Master Mix (Bio-Rad, Hercules, CA, United States) and specific primers at a final concentration of 0.9 μM (Supplementary Table 1). The program used for PCR amplification included an initial denaturation step at 95°C for 3 min, followed by 40 cycles of denaturation for 15 s at 95°C and annealing/extension for 60 s at 60°C. All the pipetting operations were made by means of an EpMotion 5070 Liquid Handling Robot (Eppendorf, Hamburg, Germany) to improve data reproducibility. The efficiency of PCRs (>92%) was checked, and the specificity of reactions was verified by analysis of melting curves (ramping rates of 0.5°C/10 s over a temperature range of 55–95°C) and linearity of serial dilutions of RT reactions (>0.99). Fluorescence data acquired during the extension phase were normalized by the delta-delta C_T_ method (Livak and Schmittgen, 2001) using *actb* in the liver, white muscle and heart, or *cox4a* in total blood cells, as the housekeeping gene due to its stability among different experimental conditions (average C_T_ varied less than 0.5 in each tissue). For multi-gene analysis, data on gene expression were in reference to the expression level of *cs* in the liver, *igfr2* in the white muscle, *gcr* in the heart, and *tim8a* in total blood cells of LDN fish, for which a value of 1 was arbitrarily assigned (Supplementary Tables 2–5, respectively).

**Table 1:** Genes included in the liver (L), white muscle (M), heart (H), and total blood cells (B) pathway-focused PCR arrays.

Gene name/category	Symbol	Gene name/category	Symbol
**GH/IGF system**		**Lipid metabolism**	
Growth hormone receptor I	*ghr-i* LMH	Elongation of very long chain fatty acids 1	*elovl1* L
Growth hormone receptor II	*ghr-ii* LMH	Elongation of very long chain fatty acids 4	*elovl4* L
Insulin-like growth factor-I	*igf-i* LMH	Elongation of very long chain fatty acids 5	*elovl5* L
Insulin-like growth factor-II	*igf-ii* LMH	Elongation of very long chain fatty acids 6	*elovl6* L
Insulin-like growth factor binding protein 1a	*igfbp1a* L	Fatty acid desaturase 2	*fads2* L
Insulin-like growth factor binding protein 2b	*igfbp2b* L	Stearoyl-CoA desaturase 1a	*scd1a* L
Insulin-like growth factor binding protein 3	*igfbp3* M	Stearoyl-CoA desaturase 1b	*scd1b* L
Insulin-like growth factor binding protein 4	*igfbp4* L	Lipoprotein lipase	*lpl* L
Insulin-like growth factor binding protein 5b	*igfbp5b* M	Peroxisome proliferator-activated receptor α	*pparα* L
Insulin-like growth factor binding protein 6b	*igfbp6b* M	Peroxisomeproliferator-activated receptor γ	*pparγ* L
Insulin receptor	*insr* M		
Insulin-like growth factor receptor I	*igfr1* M	**Antioxidant defense and tissue repair**	
Insulin-like growth factor receptor II	*igfr2* M	Catalase	*cat* LMH
		Glutathione peroxidase 4	*gpx4* LMH
**Energy sensing and oxidative metabolism**		Glutathione reductase	*gr* LMH
Sirtuin 1	*sirt1* LMH	Peroxiredoxin 3	*prdx3* LMHB
Sirtuin 2	*sirt2* LMH	Peroxiredoxin 5	*prdx5* LMHB
Sirtuin 3	*sirt3* LMH	Superoxide dismutase [Mn]	*Mn-sod/sod2* LMHB
Sirtuin 4	*sirt4* LMH	Glucose-regulated protein, 170 kDa	*grp-170* LMH
Sirtuin 5	*sirt5* LMH	Glucose-regulated protein, 94 kDa	*grp-94* LMH
Sirtuin 6	*sirt6* LMH	70 kDa heat shock protein, mitochondrial	*mthsp70/grp-75/mortalin* LMH
Sirtuin 7	*sirt7* LMH	Glutathione S-transferase 3	*gst3* B
Carnitine palmitoyltransferase 1A	*cpt1a* LMHB		
Citrate synthase	*cs* LMHB	**Muscle growth and cell differentiation**	
Proliferator-activated receptor gamma coactivator 1 alpha	*pgc1α* LMH	Myoblast determination protein 1	*myod1* M
Proliferator-activated receptor gamma coactivator 1 beta	*pgc1β* LMHB	Myogenic factor MYOD2	*myod2* M
Hypoxia inducible factor-1 alpha	*hif-1α* LMH	Myogenic factor 5	*myf5* M
		Myogenic factor 6	*myf6/mrf4/ herculin* M
**Respiration uncoupling**		Myostatin/Growth differentiation factor 8	*mstn/gdf-8* M
Uncoupling protein 1	*ucp1* L	Myocyte-specific enhancer factor 2A	*mef2a* M
Uncoupling protein 2	*ucp2* BH	Myocyte-specific enhancer factor 2C	*mef2c* M
Uncoupling protein 3	*ucp3* M	Follistatin	*fst* M
			
**Xenobiotic metabolism**		**Nuclear receptors**	
Aryl hydrocarbon receptor 1	*ahr1* H	Glucocorticoid receptor	*gcr* H
Cytochrome P450 1A1	*cyp1a1* H	Estrogen receptor alpha	*er-α* H
		Nuclear respiratory factor 1	*nrf1* B
**Outer and Inner transmembrane translocation (TOM and TIM complex)**			
Mitochondrial import receptor subunit Tom70	*tom70* B	**Mitochondrial dynamics and apoptosis**	
Mitochondrial import receptor subunit Tom34	*tom34* B	Mitofusin 2	*mfn2* B
Mitochondrial import receptor subunit Tom22	*tom22* B	Mitochondrial fission factor homolog B	*miffb* B
Mitochondrial import inner membrane translocase subunit 44	*tim44* B	Mitochondrial Rho GTPase 1	*miro1a* B
Mitochondrial import inner membrane translocase subunit 23	*tim23* B	Mitochondrial Rho GTPase 2	*miro2* B
Mitochondrial import inner membrane translocase subunit Tim8A	*tim8a* B	Apoptosis-related protein 1	*aifm1* B
Mitochondrial import inner membrane translocase subunit Tim10	*tim10* B		
Mitochondrial import inner membrane translocase subunit Tim9	*tim9* B	**OXPHOS (Complex IV)**	
		Cytochrome c oxidase subunit I	*coxi* LMHB
		Cytochrome c oxidase subunit II	*coxii* LMHB
**OXPHOS (Complex I)**		Cytochrome c oxidase subunit III	*coxiii* B
NADH-ubiquinone oxidoreductase chain 2	*nd2* LMHB	Cytochrome c oxidase subunit 4 isoform 1	*cox4a* B
NADH-ubiquinone oxidoreductase chain 5	*nd5* LMHB	Cytochrome c oxidase subunit 5A, mitochondrial-like isoform 2	*cox5a2* B
NADH dehydrogenase [ubiquinone] 1 alpha subcomplex subunit 1	*ndufa1* B	Cytochrome c oxidase subunit 6A isoform 2	*cox6a2* B
NADH dehydrogenase [ubiquinone] 1 alpha subcomplex subunit 3	*ndufa3* B	Cytochrome c oxidase subunit 6C1	*cox6c1* B
NADH dehydrogenase [ubiquinone] 1 alpha subcomplex subunit 4	*ndufa4* B	Cytochrome c oxidase subunit 7B	*cox7b* B
NADH dehydrogenase [ubiquinone] 1 alpha subcomplex subunit 7	*ndufa7* B	Cytochrome c oxidase subunit 8B	*cox8b* B
NADH dehydrogenase [ubiquinone] 1 beta subcomplex subunit 5	*ndufb5* B	SCO1 protein homolog, mitochondrial	*sco1* B
NADH dehydrogenase iron-sulfur protein 2	*ndufs2* B	Surfeit locus protein 1	*surf1* B
NADH dehydrogenase iron-sulfur protein 7	*ndufs7* B	Cytochrome c oxidase assembly protein COX15 homolog	*cox15* B
NADH dehydrogenase (ubiquinone) 1 alpha subcomplex, assembly factor 2	*ndufaf2* B		


This manuscript follows the ZFIN Zebrafish Nomenclature Guidelines for gene and protein names and symbols^1^.

### Statistical Analysis

Data on growth performance, blood biochemistry, and gene expression were analyzed by two-way analysis of variance (ANOVA) with O_2_ levels (normoxia and moderate hypoxia) and stocking conditions (low and high stocking densities) as main factors. These analyses were followed by the SNK *post hoc* test for comparisons among different groups. The significance level was set at *P* < 0.05. Analyses were performed using SigmaPlot v13 (Systat Software Inc., San Jose, CA, United States). Unsupervised multivariate analysis principal component analysis (PCA) was first performed on data as an unbiased method to observe trends within conditions at different experimental groups, using EZinfo v3.0 (Umetrics, Umeå, Sweden). To achieve the maximum separation among experimental groups, Partial Least-Squares Discriminant Analysis (PLS-DA) was sequentially applied with joint data from liver, heart and white muscle, excluding the results from total blood cells due to its low contribution to the total variance. The quality of the PLS-DA model was evaluated by R2Y(cum) and Q2Y(cum) parameters, which indicated the fitness and prediction ability, respectively. Additionally, 200 random permutation tests to avoid over-fitting of the supervised model were carried out by SIMCA-P+ v15.0 (Umetrics). Cross-validation analysis of variance (CV-ANOVA) was applied (*p*-value = 0.037). The contribution of differential genes along liver, white muscle and heart tissues was assessed by means of Variable Importance in Projection (VIP) measurements. A VIP score > 1.1 was considered to be an adequate threshold to determine discriminant variables in the PLS-DA model (Wold et al., 2001; Li et al., 2012; Kieffer et al., 2016).

## Results

### Growth Performance

Data on feed intake, growth and somatic indexes (hepatosomatic index, HSI; viscerosomatic index, VSI) are shown in Table 2. Fish reared under moderate hypoxia evidenced lower feed intake, which resulted in reduced weight gain and SGR. This also affected liver and viscera weight as well as HSI. This general impairment of feed intake and growth was further evidenced in fish kept at the highest density, though FE was improved in moderate hypoxia and more especially in fish kept at HD (HDH group).

**Table 2:** Effects of rearing density and dissolved oxygen level on gilthead sea bream growth performance on a 21-days feeding trial.

	LD		HD		*P*-value		
			
	Normoxia	Hypoxia	Normoxia	Hypoxia	[O_2_]	Density	Interaction
Initial body weight (g)	34.54 ± 1.11	34.22 ± 0.27	34.32 ± 0.34	33.25 ± 0.45	n.s.	n.s.	n.s.
Final body weight (g)	56.04 ± 1.89	51.65 ± 0.71	54.02 ± 0.50	48.54 ± 1.05^**^	0.003	n.s.	n.s.
Feed intake (g DM/fish)	23.78 ± 1.63	18.52 ± 0.7^*^	24.57 ± 1.06	17.54 ± 0.47^**^	< 0.001	n.s.	n.s.
Weight gain (%)^1^	62.21 ± 0.31	50.94 ± 1.34^**^	57.43 ± 1.42	45.97 ± 1.31^**^	< 0.001	0.003	n.s.
SGR (%)^2^	2.30 ± 0.01	1.96 ± 0.04^**^	2.16 ± 0.04	1.80 ± 0.04^**^	< 0.001	0.004	n.s.
FE (%)^3^	0.91 ± 0.03	0.94 ± 0.02	0.80 ± 0.02	0.87 ± 0.01^*^	0.039	0.003	n.s.
Liver weight (g)	0.94 ± 0.07	0.67 ± 0.03^**^	0.90 ± 0.06	0.63 ± 0.03^***^	< 0.001	n.s.	n.s.
Viscera weight (g)	4.41 ± 0.28	3.84 ± 0.18	4.42 ± 0.19	3.68 ± 0.10^**^	0.002	n.s.	n.s.
HSI (%)^4^	1.64 ± 0.07	1.33 ± 0.06^**^	1.58 ± 0.07	1.25 ± 0.06^**^	< 0.001	n.s.	n.s.
VSI (%)^5^	7.78 ± 0.29	7.65 ± 0.25	7.87 ± 0.24	7.38 ± 0.22	n.s.	n.s.	n.s.


*Values on body weight, feed intake, growth, and feed efficiency are the mean ± SEM of triplicate tanks. Values on tissue biometric indexes are the mean ± SEM of 12 fish (4 fish per replicate tank). *P*-values are the result of two-way analysis of variance. Non-significance (*P* > 0.05) is stated by “n.s.”. Asterisks in each row indicate significant differences with oxygen level for a given rearing density (SNK test, *P* < 0.05). ^*1*^Weight gain (%) = (100 × body weigh increase)/initial body weight. ^*2*^Specific growth rate = 100 × (ln final body weight – ln initial body weight)/days. ^*3*^Feed efficiency = wet weight gain/dry feed intake. ^*4*^Hepatosomatic index = (100 × liver weight)/fish weight. ^*5*^Viscerosomatix index = (100 × viscera weight)/fish weight.*

### Blood Analysis

Data on blood hematology and biochemistry are shown in Table 3. The results show a significant effect of O_2_ level, with a generalized increase in Hb, Ht, RBC content, MCH, cortisol and Gh plasma levels, as well as a widespread decrease in MCHC, MCV and plasma lactate levels. Overall this feature was more pronounced in fish maintained under LD conditions. In contrast, the rearing density effect was mostly evidenced in plasma cortisol levels, which showed a pronounced rise in HD fish that was exacerbated by hypoxic conditions. Noticeably, significant O_2_ level and rearing density interactions were found for cortisol, but also for Ht, MCHC, MCH, and TAA.

**Table 3:** Effects of rearing density and dissolved oxygen level on blood hematology and plasma levels of metabolites, hormones, and total antioxidant capacity.

	LD		HD		*P*-value		
			
	Normoxia	Hypoxia	Normoxia	Hypoxia	[O_2_]	Density	Interaction
Hemoglobin (g/dl)	7.18 ± 0.24	7.73 ± 0.21	7.38 ± 0.14	7.77 ± 0.26	0.041	n.s.	n.s.
Haematocrit (%)	22.18 ± 1.10	32.91 ± 1.65^***^	28.27 ± 1.77	29.90 ± 1.39	< 0.001	n.s.	0.004
RBC× 10^-6^ (cells/μl)^1^	2.45 ± 0.07	2.74 ± 0.07^**^	2.38 ± 0.06	2.82 ± 0.08^***^	< 0.001	n.s.	n.s.
MCHC (pg/10 μm^3^)^2^	34.07 ± 1.12	24.00 ± 1.18^***^	26.62 ± 1.73	26.46 ± 1.10	< 0.001	n.s.	< 0.001
MCH (pg/cell)^3^	89.79 ± 4.21	116.6 ± 4.46^**^	116.5 ± 8.28	109.5 ± 7.21	n.s.	n.s.	0.010
MCV (μm^3^)^4^	29.50 ± 1.02	28.33 ± 0.76	31.36 ± 0.93	27.73 ± 0.96^*^	0.014	n.s.	n.s.
Glucose (mg/dl)	54.39 ± 1.58	52.17 ± 2.44	58.04 ± 1.78	52.73 ± 2.79	n.s.	n.s.	n.s.
Lactate (mg/dl)	16.30 ± 2.78	4.81 ± 1.41^**^	10.22 ± 3.06	4.99 ± 0.84	0.001	n.s.	n.s.
TAA (mM Trolox)^5^	1.34 ± 0.04	1.45 ± 0.04	1.48 ± 0.03	1.43 ± 0.03	n.s.	n.s.	0.026
Cortisol (ng/ml)	23.40 ± 5.67	21.08 ± 5.32	35.69 ± 11.15	79.25 ± 9.05^**^	0.036	< 0.001	0.027
Growth hormone (ng/ml)	2.34 ± 0.83	6.71 ± 1.17^*^	5.39 ± 1.29	8.33 ± 4.20	n.s.	n.s.	n.s.
Insulin-like growth factor-I (ng/ml)	46.06 ± 4.76	46.59 ± 4.77	45.78 ± 2.27	41.03 ± 6.29	n.s.	n.s.	n.s.


*Values are the mean ± SEM of 10–12 fish (4 fish per replicate tank). Non-significance (*P* > 0.05) is stated by “n.s.”. *P*-values are the result of two-way analysis of variance. Asterisks in each row indicate significant differences with oxygen level for a given rearing density (SNK test, *P* < 0.05). ^*1*^Red blood cells ^*2*^Mean corpuscular hemoglobin concentration. ^*3*^Mean corpuscular hemoglobin. ^*4*^Mean corpuscular volume. ^*5*^Total antioxidant activity.*

### Gene Expression Profiling

All genes selected for PCR-arrays were found at detectable levels in the four tissues analyzed. Results of gene expression profiling in hepatic selected genes are presented in Supplementary Table 2. Among them, 22 out of 43 genes were affected by at least one of the experimental factors or by its interaction (i.e., the combined effect of confinement stress and hypoxia exposure leading to expression changes that could not be attributed to a single parameter), being 11 differentially expressed (DE) in response to O_2_ level. Relative expression of markers from GH/IGF system (*ghr-i*), oxidative metabolism (*nd2*), and antioxidant defense and tissue repair (*gpx4, prdx5*) was significantly down-regulated by moderate hypoxia in LDH and HDH groups. In addition, several genes of lipid metabolism (*elovl1, fads2*, and *scd1b*) were up-regulated in the LD group, whereas markers of oxidative metabolism (*nd5*), and antioxidant defense and tissue repair (*gr, sod2*, and *grp-75*) were down-regulated in fish kept at HD conditions. Stocking density also affected 11 genes related with the GH/IGF system (*ghr-i, ghr-ii*, and *igf-i*), lipid metabolism (*elovl6, fads2, scd1a*, s*cd1b*, and *lpl*), oxidative metabolism (*ucp1, pgc1*α) and antioxidant defense and tissue repair (*grp-75*). A statistically significant interaction of O_2_ levels and rearing density was found for *igf-ii, fads2, scd1a, scd1b, pgc1ß, gr, prdx3*, and *grp-170* genes.

In white skeletal muscle, 20 out of 44 DE genes were affected at least by one of the experimental condition or by their interaction (Supplementary Table 3). Markers of the GH/IGF system were mostly affected by stocking density (*ghr-i, igf-ii, igfbp3, igfbp5b, igfbp6b, insr*, and *igfr1*) rather than by O_2_ levels (*igfr2*). Moderate hypoxia up-regulated *myod2* expression as the sole effect on genes related to muscle growth and cell differentiation. In contrast, many genes related to energy sensing, oxidative metabolism, and antioxidant defense and tissue repair were down-regulated by low O_2_ levels (*sirt1, ucp3, hif-1*α, *prdx5*, and *sod2*) or up-regulated in HD conditions (*sirt4, sirt7, coxi, hif-1*α, and *gpx4*). Additionally, a significant interaction between O_2_ levels and rearing density is reported for *cpt1a* and *grp-170*.

In heart, changes in O_2_ saturation and stocking density triggered significant differences in 19 out of 34 genes presented in the array (Supplementary Table 4). Up to 13 genes, including markers of the GH/IGF system (*ghr-i*), energy sensing and oxidative metabolism (*sirt1, sirt5, sirt6, sirt7, cs, nd5, pgc1*α, *pgc1ß*, and *hif-1*α) and antioxidant defense and tissue repair (*cat, prdx5*, and *sod2*) were down-regulated under moderate hypoxia, especially in HD conditions. The xenobiotic metabolism marker *cyp1a1* was up-regulated by hypoxia in both LD and HD. Stocking density also down-regulated the expression of several genes involved in energy sensing and oxidative metabolism (*sirt3, sirt5, cs*, and *nd2*) as well as antioxidant defense and tissue repair (*gr, prdx3, prdx5, grp-170*, and *grp-75*), preferentially under low O_2_ levels.

In total blood cells, only 5 out of 44 genes were DE mainly by the interaction among different experimental conditions (Supplementary Table 5), being responsive to the stress challenge enzyme subunits of Complex I (*ndufaf2*) and Complex IV (*coxi, coxii, cox6a2*, and *cox15*) of the mitochondrial respiratory chain.

**FIGURE 1:**
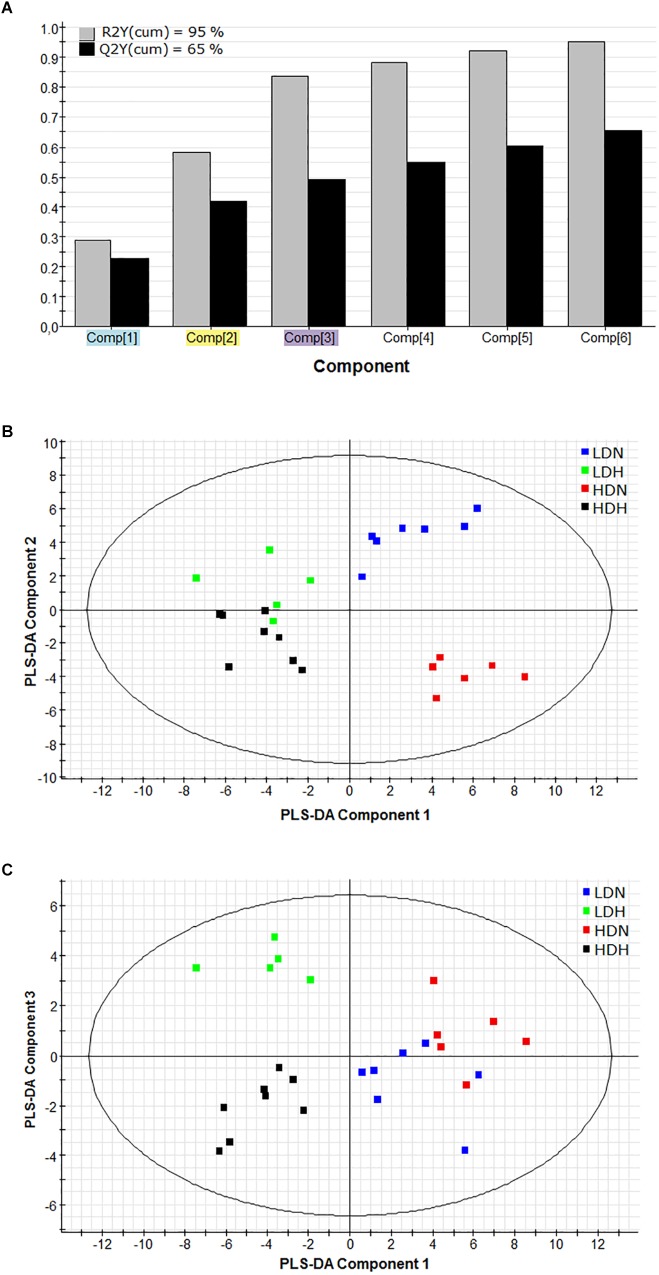
**(A)** Graphical representation of the goodness-of-fit of the PLS-DA model. **(B)** Two-dimensional PLS-DA score plot representing the distribution of the samples between the first two components in the model. **(C)** Two-dimensional PLS-DA score plot representing the distribution of the samples between the first and third components in the model. R^2^(cum), explained variance; Q^2^(cum), predicted variance; LDN, low density normoxia; LDH, low density hypoxia; HDN, high density normoxia; HDH, high density hypoxia.

In order to assess the differential contribution of the DE genes in the physiological response to moderate hypoxia and rearing density, the tissue (liver, white skeletal muscle, and heart) gene expression dataset was analyzed by PLS-DA. The discriminant model was based on six components, which explained [R2Y(cum)] 95% and predicted [Q2Y(cum)] 65% of total variance (Figure 1A). The validity of the PLS-DA model was validated using a permutation test (Supplementary Figure 2). The first three components showed cumulative values for R2Y and Q2Y of 0.836 and 0.493, respectively. A clear separation between normoxic (LDN, HDN) and hypoxic (LDH, HDH) groups was observed along the first component (R2Y = 0.2889) (Figure 1B,C). Component 2 (R2Y = 0.2927) clearly separated LDN and HDN normoxic groups (Figure 1B), whereas component 3 (R2Y = 0.2542) discriminated LDH and HDH hypoxic groups (Figure 1C).

**FIGURE 2:**
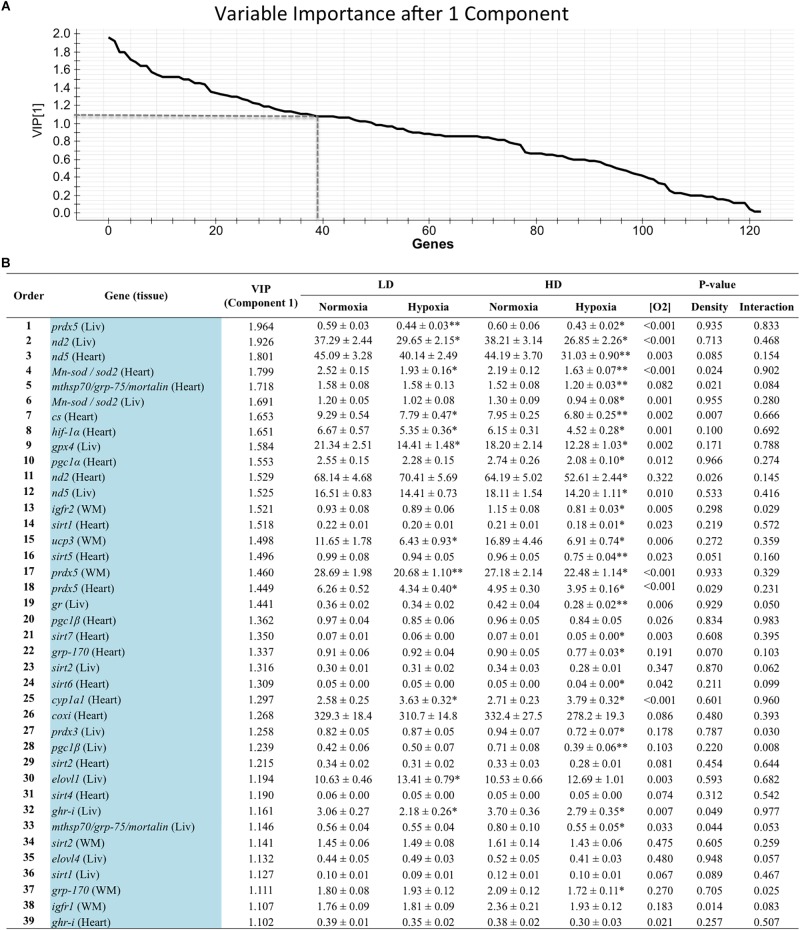
**(A)** Graphical representation of the variable importance (VIP) scores after component 1. **(B)** Ranking list of genes showing VIP score values above 1.1 and their relative gene expression. Liv, liver; WM, white muscle. Values on relative expression are the mean ± SEM of eight fish (2–3 fish per replicate tank). *P*-values are the result of two-way analysis of variance. Asterisks in each row indicate significant differences with oxygen level for a given rearing density (SNK test, *P* < 0.05).

**FIGURE 3:**
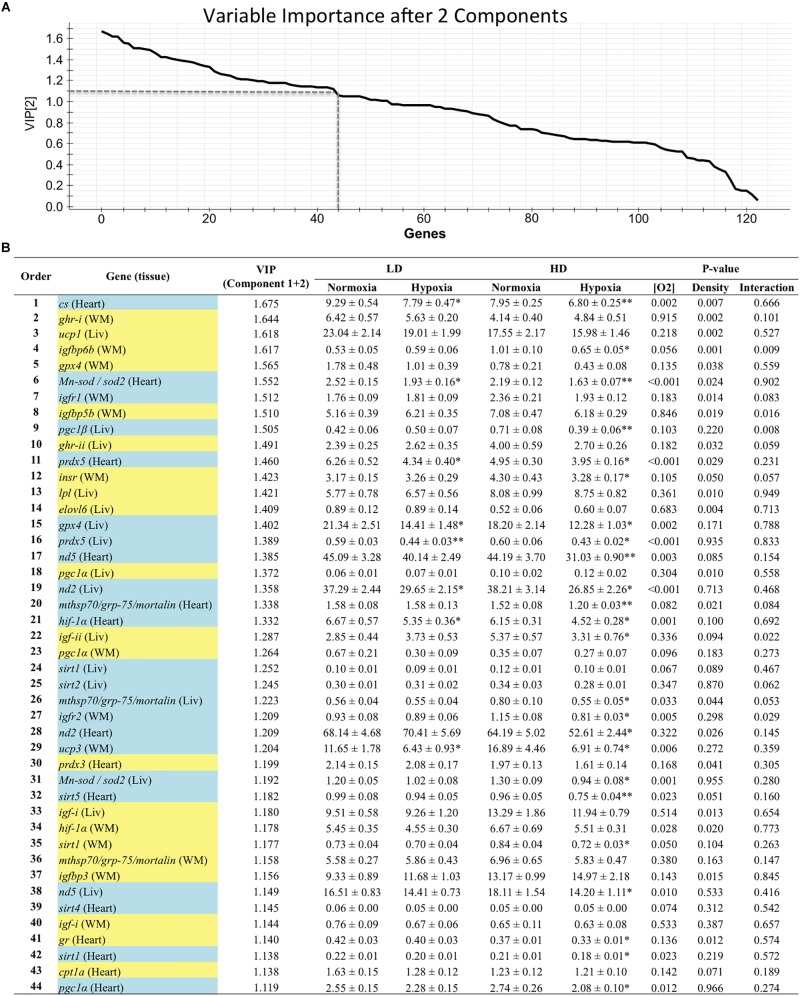
**(A)** Graphical representation of the variable importance (VIP) scores after component 2. **(B)** Ranking list of genes showing VIP score values above 1.1 and their relative gene expression. Cells shaded in blue highlight genes detected as VIP after component 1; cells shaded in yellow highlight genes detected as VIP after component 2. For further details, see legend on Figure 2.

**FIGURE 4:**
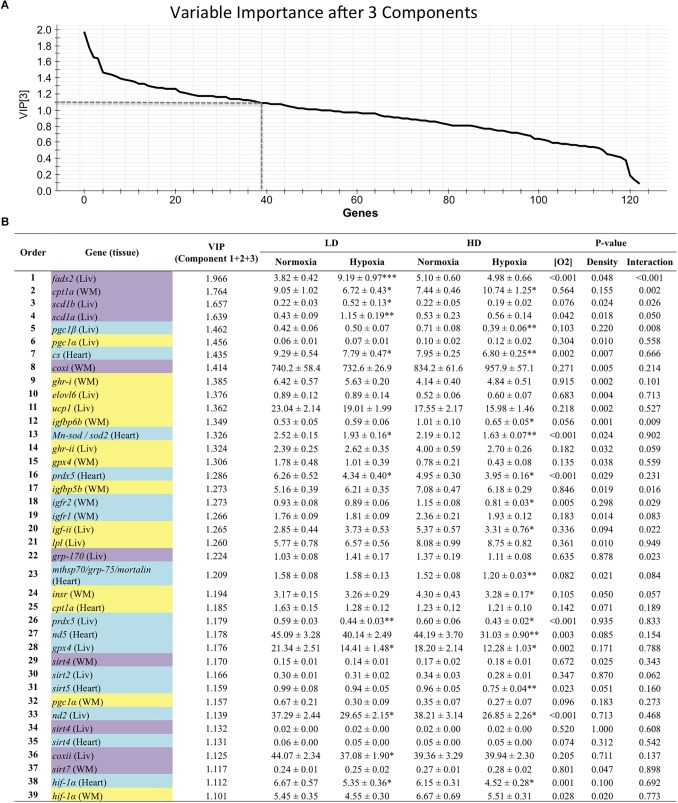
**(A)** Graphical representation of the variable importance (VIP) scores after component 3. **(B)** Ranking list of genes showing VIP score values above 1.1 and their relative gene expression. Cells shaded in blue highlight genes detected as VIP after component 1; cells shaded in yellow highlight genes detected as VIP after component 2; and cells shaded in purple highlight genes detected as VIP after component 3. For further details, see legend on Figure 2.

Genes with a contribution to VIP > 1.1 in component 1 were a total of 39, with a main contribution of heart (19) and liver (14) genes involved in energy sensing and oxidative metabolism (14), antioxidant defense and tissue repair (12) and OXPHOS (Figure 2). When the second component was also considered, a total of 44 genes presented VIP values > 1.1 (Figure 3), and 11 out of the 21 new genes (highlighted in yellow) were from white skeletal muscle. Energy sensing and oxidative metabolism (12), antioxidant defense and tissue repair (11), GH/IGF system (11) and OXPHOS (6) were the main categories. Considering the VIP values from the three main components (Figure 4), most of the genes due to component three contribution (highlighted in purple) were related to lipid metabolism.

## Discussion

Hypoxia in aquatic habitats is a common disturbance that is predicted to occur in the future more extensively, more frequently and for longer periods of time (Intergovernmental Panel on Climate Change, 2014), becoming a major aquaculture stressor around the world. This is especially true in the case of intensive fish farming, and unraveling the adaptive hypoxic responses helps to better understand the nature of metabolic disturbances after short- and long-term exposures to challenging O_2_ levels. Blood physiological landmarks remain mostly unaltered in juveniles of gilthead sea bream exposed over 24 h to moderate hypoxia (40% O_2_ saturation), whereas changes in Ht and circulating levels of Hb, glucose and lactate are reported few hours after acute hypoxia (20% O_2_ saturation) (Martos-Sitcha et al., 2017). In the same study, gene expression profiling of total blood cells evidenced a consistent transcriptional response after strong hypoxic challenges, which serve to ensure a reduced but more efficient aerobic ATP production during severe hypoxia. Herein, the combined effects of moderate hypoxia (42–43% O_2_ saturation) and rearing density (initial density 19 kg/m^3^, leading up to 30 kg/m^3^ at the end of experiment) in a 3-week trial highlighted reduced growth and a different contribution of target tissues to the homeostatic load in challenged fish. As discussed below, the ultimate mechanisms for this adaptive stress response remain far to be established, though probably they have a major impact in mitochondrial respiration uncoupling, which varies across life, tissues, individuals and species (Rolfe and Brand, 1996; Hulbert et al., 2002, 2006). Indeed, improved energy efficiency and reduced mitochondrial respiration uncoupling becomes a priority with low food availability (Auer et al., 2015), and the expression of mitochondrial uncoupling proteins (UCP2/UCP3) is differentially regulated by feed restriction in glycolytic (white skeletal muscle) and highly oxidative (heart and skeletal red muscle) tissues of gilthead sea bream (Bermejo-Nogales et al., 2014a).

Growth impairments due to long-term hypoxia exposure have been noticed in a wide-range of farmed fish, including turbot (*Scophthalmus maximus*), European sea bass (*Dicentrarchus labrax*), and Atlantic salmon (*Salmo salar*) (Pichavant et al., 2001; Remen et al., 2016; Cadiz et al., 2017; Vikeså et al., 2017). As reported herein in gilthead sea bream, a primary response is the inhibition of feed intake which would favor a hypo-metabolic state with a reduced ROS production and risk of oxidative stress. This is supported by lowered plasma levels of lactate, which would reflect in hypoxic fish, and in a lower extent in HDN, a low basal metabolism rather than a shift of aerobic to anaerobic metabolism. This metabolic re-adjustment has also been reported in gilthead sea bream juveniles facing multiple sensorial stressors in a model of chronic stress that mimic daily aquaculture operations (Bermejo-Nogales et al., 2014b). Thus, according with the oxystatic theory (Dam and Pauly, 1995; Saravanan et al., 2012), fish finely adjust feed intake and basal metabolism to available O_2_, prioritizing feed efficiency at the expenses of maximum growth under restricted mitochondrial respiration. As a prove of this, the best FE and hormonal signatures for fast and efficient growth generally occurs before the achievement of maximum growth at the greater ration size (Brett, 1979; Pérez-Sánchez et al., 1995), pointing out a high metabolic plasticity in this euryhaline, eurytherm and euryoxic fish species due to a permissive regulation of feed intake which allows to cope an efficient energy metabolism at slow growth rates. This also applies at the cellular level, where the maximum ATP yield per molecule of O_2_ (P/O ratio) is highly dependent on ration size, as evidenced the increased P/O ratio of king penguins during periods of food shortage (Monternier et al., 2014) or liver mitochondria of brown trout (*Salmo trutta*) starved for 2 weeks (Salin et al., 2016).

Most of the hypoxia-mediated effects are accompanied by an enhanced O_2_-carrying capacity denoted by a swelling, formation and/or release of new erythrocytes together with plasma volume reduction (Gallaugher and Farrell, 1998). A set of different mechanisms operate this complex response in fish. It must be considered that fish RBC are nucleated and have the ability to produce Hb during most of their life span (Speckner et al., 1989). As a general response to stress, erythrocytes from fish spleen reservoirs can be released into the blood (Pearson and Stevens, 1991). In the other hand, the affinity of fish Hb to O_2_ can be modulated by allosteric regulation (variations in phosphate levels) (Val, 2000). This ability to increase blood O_2_ affinity as a response to hypoxia is present even in fish living in well-oxygenated environments, as it is the case of the Antarctic fish bald rockcod (*Pathogenia borchgrevinki*) (Wells et al., 1989), a finding that highlights the general phenotypic plasticity of fish. Hypoxia studies on rainbow trout (*Oncorhynchus mykiss*) showed an initial increase in Hb concentration mediated by the release of spleen erythrocytes, but under persistent hypoxia conditions the increase of the O_2_-carrying capacity arose from synthesis of new erythrocytes (Lai et al., 2006). These results suggest a complex and dynamic adaptation of fish to hypoxic conditions, a feature that could be species-specific. From our hematological data in the present work, hypoxia induced a slight increase of Hb content at both rearing densities, although the most evident and significant effects were the increase of the measured Ht, RBC count and corpuscular concentrations of Hb, which were secondly affected by rearing density. A similar enhancement of O_2_-carrying capacity by means of Ht increase was observed not only in previous short-term acute hypoxia challenges in gilthead sea bream (Magnoni et al., 2017; Martos-Sitcha et al., 2017), but also in eels and rainbow trout (Wood and Johansen, 1972; Soivio et al., 1980). Conversely, changes in HSI, reflecting the amount of lipid and glycogen depots, are more informative of feed intake rather that hypoxic condition, though it is difficult to disclose the main factor. At the hormonal level, this is also inferred from the measurements of circulating levels of cortisol and Gh, which are well-known regulators of metabolic rates by their involvement on mitochondria function (see Mommsen et al., 1999; Bergan-Roller and Sheridan, 2018 for review).

In fact, cortisol is an established marker of crowding stress in gilthead sea bream (Arends et al., 1999; Skrzynska et al., 2018), as well as under other challenging conditions, and the reduced feed intake as a consequence of the stress challenge also enhanced the responsiveness of the hypothalamic-pituitary-adrenal axis (Sangiao-Alvarellos et al., 2005). Thus, cortisol is the main corticosteroid in teleost fish and its plasma levels can increase dramatically during unfavorable situations activating specific intracellular responses through glucocorticoid receptors present in many fish organs, which in turn alleviate the great energy demand in a systemic way by increasing blood metabolite concentrations and redistributing the energy balance in the organism (reviewed by Mosconi et al., 2006; Wendelaar Bonga, 2011). In fish, high plasma cortisol levels modulate the metabolism of carbohydrates by stimulating gluconeogenesis in liver, and also increase the availability of substrates derived from proteins and fats, although the role of cortisol in fish lipid metabolism has not been clearly established (Vijayan et al., 2010). All this agrees with the observation that the greater circulating concentration of cortisol was achieved herein in the HDH group, which also experienced a higher feed intake inhibition. However, this system cannot be continuously refed, and evidence in rodents points out toward a translocation of cortisol into mitochondria mediated by glucocorticoid receptors to reduce mitochondrial activity and the risk of oxidative stress (Du et al., 2009), a mechanism that could be possibly extended to other animal models including fish, although this fact has still not been documented. Thus, in the absence of a cortisol response, chronic cold-thermal stress up-regulates OXPHOS in gilthead sea bream, whereas the cortisol rise in fish facing multiple aquaculture sensorial stressors is accompanied by a pronounced transcriptional repression of all the hepatic complex units of the mitochondrial respiratory chain (Bermejo-Nogales et al., 2014b). A similar response has been reported after acute hypoxia exposure, though in this case the catalytic and regulatory enzyme subunits of Complex IV (the last electron acceptor of respiratory chain involved in the O_2_ reduction) were up-regulated, maximizing the use of available O_2_ for aerobic ATP generation (Martos-Sitcha et al., 2017). The aerobic scope and gene expression profiling of mitochondria is also highly regulated at the nutritional level by synthetic and natural dietary oils (Pérez-Sánchez et al., 2013; Martos-Sitcha et al., 2018), and the suppression of heptanoate effects upon exercise endurance is viewed as a protective measure to counteract disproportionate oxidative metabolic rates in fish fed fast energy-delivery nutrients (short/medium chain fatty acids). In other words, the interplay between stimulatory and inhibitory effects must be envisaged as a response to the energy needs, even if metabolic fuels were available. Accordingly, in the present study, the increased circulating levels of Gh in hypoxic/crowded fish will reflect a reduced feed intake and energy demand rather than a minor capacity to combat oxidative stress, as it is generally referenced in fish and other animal models overexpressing GH (Brown-Borg et al., 1999; Brown-Borg and Rakoczy, 2000; McKenzie et al., 2003; Almeida et al., 2013).

The gene expression profiling of key metabolic biomarkers also contributes to better understand the search of allostatic load in a challenging environment. Thus, the two-way ANOVA revealed the different involvement of tissues and gene categories into the stress-mediated responses. This observation is reinforced by the use of multivariate analysis, which offers the possibility to identify, at a high level of confidence, the most responsive tissues and biomarkers for a given stress stimuli in a factorial stress design. Using such approach, we are able to explain and to predict a high percentage of total variance, being noteworthy that liver, white skeletal muscle and heart remained responsive at long-term to changing O_2_ and rearing density, whereas the expression pattern of blood cells became mostly unaltered with the imposed stress stimuli of medium intensity, in contrast with the previously reported wide expression change of mitochondrial-related genes in total blood cells in response to a more severe challenge (Martos-Sitcha et al., 2017). For this reason, the transcriptomic analysis of total blood cells during moderate hypoxia challenges was not included in our PLS-DA model to avoid the background noise detected when introduced. In previous studies in gilthead sea bream and other animal models, liver and cardiac muscle are highly responsive to hypoxia (Everett et al., 2012; Hermes-Lima et al., 2015; Magnoni et al., 2017), and genes of these two tissues highly contributed herein to separate normoxic and hypoxic fish along the first component of our PLS-DA (R2Y = 0.2889). One of the most relevant genes participating in this discriminant feature is the *hif-1*α, a well-documented regulator of O_2_ homeostasis. This transcriptional factor acts at a multi-regulatory level, managing the hypoxic responsiveness of a vast array of transcribed proteins including antioxidant enzymes (Nikinmaa and Rees, 2005; Lushchak and Bagnyukova, 2006). Concretely, herein, we show a clear down-regulation of *hif-1*α that was coincident with the repressed expression of other down-stream markers of antioxidant defense and tissue repair (*prdx5, sod2, mortalin, gpx4, gr, grp-170*, and *prdx3*). This intriguing result can be cautiously interpreted since Hif-1 is mostly regulated at the post-translational level (Ke and Costa, 2006), though this finding should be understood as a steady-state in which O_2_ availability is reduced but maintained high enough to preserve aerobic metabolism at a relatively high level. This fact is supported by a reduced expression of *cs* and associated enzyme subunits of Complex I (*nd2, nd5*), used successfully in several studies as markers of mitochondria abundance and Krebs cycle activity (Larsen et al., 2012; Magnoni et al., 2017). In addition to that, several *sirts* (*sirt1, 2, 5, 6*, and *7*) of liver or cardiac muscle were overall down-regulated in hypoxic fish, especially in the case of HDH fish. These NAD^+^-dependent deacetylases are energy sensors that act in gilthead sea bream as a link between nutrition and energy metabolism in different growth models with nutrients and genetic variables as source of variation (Simó-Mirabet et al., 2017a,b, 2018). This was extended herein to hypoxia/crowding stress, which indicates that most of the envisaged adaptive responses should include changes in the acetylation status of both nuclear histones, and cytoplasmic and mitochondrial metabolic enzymes.

The second component of our PLS-DA (R2Y = 0.2927) differentiates normoxic fish held at different stocking densities. In this case, the white skeletal muscle clearly promotes this separation mainly by the expression pattern of genes related to GH/IGF system (*ghr-i, igfbp6b, igfbp5b, insr, igfbp3, igf-i*). Components of liver and muscle GH/IGF system are differentially regulated by nutrients and seasonal environmental cues (reviewed by Pérez-Sánchez et al., 2018), but herein this observation becomes specially relevant for muscle *ghr-i* that highly contributes to discriminate the detrimental growth effects of crowding stress from those more related to hypoxia or water quality. Likewise, genes of *igfbp* repertoire highly contribute to this differentiation, though the discriminant role of Igfbp counterparts (*igfbp6b > igfpb5b > igfpb3)* was mostly reduced to skeletal muscle and *Igfbp3/5/6* clade. Functional divergence regarding the growth-inhibitory or growth-promoting action of *igfbp*s have been reported across species and physiological context (Garcia de la Serrana and Macqueen, 2018), but herein the overall depressed expression of the muscle *Igfbp* clade in HD fish is consistent with inhibitory rather than stimulatory growth-promoting effects, which also involves the regulation of insulin and Igfbp receptors with important implications on the final arrangements of carbohydrate, growth, and energy metabolism (reviewed by Reindl and Sheridan, 2012; Vélez et al., 2017). Indeed, fish are the first group in the vertebrate tree in which there is evidence of distinct insulin and Igf receptors, though certain cross-reactivity between ligand and receptors of insulin and Ifgs occurs and the specific-mediated effects are sometimes confounding. However, it is well-recognized that insulin stimulates Hif-1, whereas intermittent hypoxia induces insulin resistance in mice (Treins et al., 2002; Poulain et al., 2017). Likewise, *Igfbp1* knock-down alleviates the hypoxia-induced growth retardation in zebrafish (Kajimura et al., 2005), whereas the IGFBP4 expression is induced by hypoxia in U87 glioma cells (Minchenko et al., 2016). From our results it is also conclusive that the muscle expression of *igfr1 and igfr2* are specially responsive to hypoxia, but importantly *insr* in gilthead sea bream seems to be more receptive to crowding stress rather than hypoxic stress stimuli, though it remains to be established the functional relevance of this differential responsiveness to environmental stressors.

Finally, the third component of our multivariate approach (R2Y = 0.2542) discriminates the effect of stocking density in fish exposed to moderate hypoxia, with a marked contribution of hepatic fatty desaturases with Δ6 (*fads2*) or Δ9 (*scd1a, scd1b*) activities due to its strong and specific induction in LDH fish. A muscle marker of FA oxidation (*cpt1a*) was also consistently up-regulated in this group, but this response was opposite to that found in HDH group, which is indicative of the different regulation of muscle lipid catabolism by hypoxia in fish stocked at standard or high densities. Likewise, the major discriminant capacity of other factors related to lipid metabolism (*elovl6*) was achieved between normoxic fish held at LD and HD. Meanwhile, other elongases (*elovl5*) with a well-recognized role in the control of hepatic triglyceride storage did not take part of the group separation in the present study, though *elovl5* highly contributes to differentiate two gilthead sea bream strains with differences in growth performance and metabolic capacities (Simó-Mirabet et al., 2018). Previous studies, in gilthead sea bream (Benedito-Palos et al., 2013, 2014) and European sea bass (Rimoldi et al., 2016) have also evidenced an important effect of ration size on the hepatic and muscle regulation of most of the lipid biomarkers assessed in the present study, but again it is difficult to disclose what is the main factor (feed intake or the imposed stress condition) due to the logistic limitations of our experiment design that did not include pair-fed groups. However, as a general rule, stressors enhance the demand of specific nutrients and hypoxia in particular promote the cellular uptake of extracellular unsaturated fatty acids in mice cell lines (Ackerman et al., 2018). Moreover, in hypoxic stress, cancer cells enhance lipid synthesis that is important for membrane biosynthesis and energy storage for cell survival and proliferation (Huang et al., 2014), being induced this hypoxia lipogenic phenotype via dependent- and HIF1α-independent pathways (Valli et al., 2015). All this together supports the pronounced stimulation of *fads2* and *scd* desaturases in our stress model, which will promote the increase of the unsaturation index of structural lipids as previously reported during feed restriction in gilthead sea bream (Benedito-Palos et al., 2013). In agreement with this, hypoxia stress on HeLa cells leads to significant changes in their membrane lipid profiles, and polyunsaturated phospholipid species are becoming stronger biomarkers for discriminating the effect of hypoxia treatment on membrane fluidity and further membrane-dependent functions (Yu et al., 2014).

A growing effort is devoted in fish to define a “stressome,” or a catalog of genes expressed when an organism is challenged with a given stress, particularly those that comprise a common response to diverse stressful scenarios (as reviewed in Balasch and Tort, 2019). Our work follows a similar approach in order to determine not only the most consistent and reliable biomarkers for welfare assessment, but also the most (or least) convenient tissues for these analyses.

## Concluding Remarks

The findings described herein evidence the re-adjustment of several biological functions in a factorial model of chronic stress, where most of the hypoxia-mediated effects on growth performance and energy metabolism were exacerbated in fish held at HD. The integrated data on blood hematology, biochemistry and hormonal profiling highlights a hypo-metabolic state with the enhancement of O_2_-carrying capacity, being this metabolic feature accompanied by a reduction in voluntary feed intake and a more efficient energy metabolism at the expenses of slow growth rates. This notion was supported at the transcriptional level by global changes of tissue-gene expression profiles, which also evidenced tissue-specific orchestration of stress response reflecting the nature and intensity of stress stimuli, but also the different metabolic capacities of targeted tissues. Thus, the number of DE in response to a given stress stimuli varies across the targeted tissues (liver ≥ heart > muscle > blood), but importantly PLS-DA analysis also informs of the different tissue contribution to the allostatic load. Thus, liver and heart mostly contribute to cope with a global hypoxic response involving changes in energy sensing and production as well as antioxidant defense and tissue repair. In contrast, metabolic markers of skeletal muscle with a high over-representation of GH/IGF system mostly contribute to disclose the effects of rearing density not necessarily mediated by low O_2_ levels. Likewise, lipid metabolism and hepatic fatty acid desaturases are becoming strong biomarkers of crowding stress in hypoxic fish, which reveals the complexity and metabolic plasticity of gilthead sea bream to cope with stress resilience under intensive fish farming. These results evidence the potential of the identified biomarkers for a reliable assessment of fish welfare, although for some tissues such as blood cells, responsiveness is highly dependent on the intensity of the challenge. Overall, this new knowledge will contribute to better explain and understand the different stress resilience of farmed fish across individuals and species.

## Ethics Statement

All procedures decribed here were approved by the Ethics and Animal Welfare Committee of Institute of Aquaculture Torre de la Sal and carried out according to national (Royal Decree RD53/2013), and the EU legislation (2010/63/EU) on the handling of animals for experiments.

## Author Contributions

JM-S, JC-G, and JP-S conceived and designed the study. JM-S, PS-M, and VdlH carried out the experimental procedures. JM-S and JP-S wrote the original draft. All authors analyzed and interpreted the data, reviewed, edited, and approved the final manuscript.

## Conflict of Interest Statement

The authors declare that the research was conducted in the absence of any commercial or financial relationships that could be construed as a potential conflict of interest.

## Footnotes

1 https://wiki.zfin.org/display/general/ZFIN+Zebrafish+Nomenclature+Conventions
