# Supplementary material for: Tissue-Specific Orchestration of Gilthead Sea Bream Resilience to Hypoxia and High Stocking Density
Source: Front Physiol. 2019 Jul 10;10:840. doi: 10.3389/fphys.2019.00840 (PMC6635561; doi:10.3389/fphys.2019.00840)
Supplement: Supplementary file 1 [file Image_1.pdf]

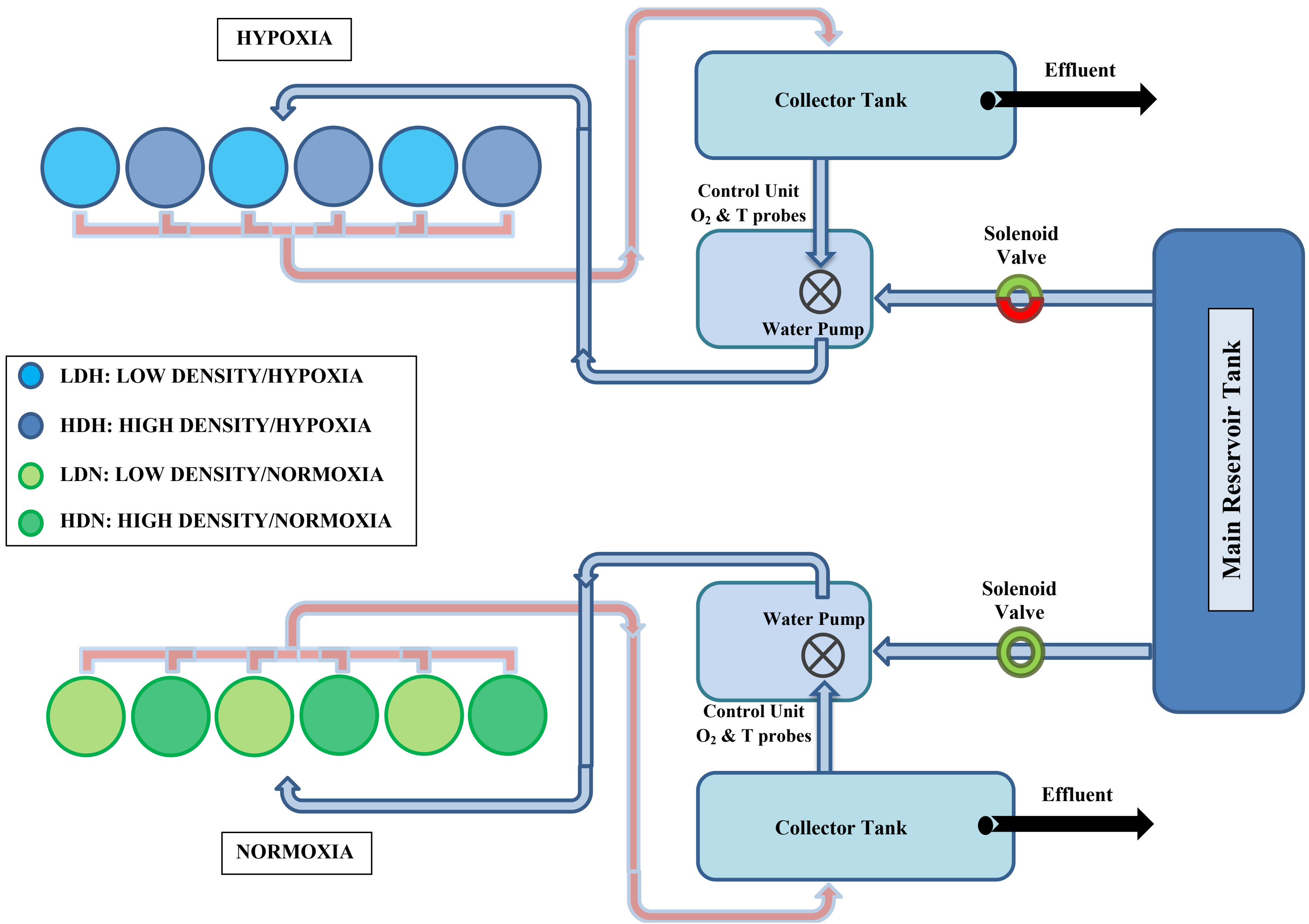

Supplemental Figure 1. Experimental set-up used to control dissolved O_2 levels in the experimental tanks to implement hypoxic conditions under different stocking densities. The steady-state was set at ~3.0 ppm O_2 (42-43% oxygen saturation) in fish kept under hypoxia (LOS), whereas fish maintained in normoxia a concentration of >5.5 ppm O_2 (>85 % O_2 saturation) was always assured.
