# Supplementary material for: Tissue-Specific Orchestration of Gilthead Sea Bream Resilience to Hypoxia and High Stocking Density
Source: Front Physiol. 2019 Jul 10;10:840. doi: 10.3389/fphys.2019.00840 (PMC6635561; doi:10.3389/fphys.2019.00840)
Supplement: Supplementary file 3 [file Table_1.docx]

**Suppl. Table 1.** Forward (F) and reverse (R) primers used for real-time PCR.

| **Gene name** | **Symbol** | **Acc. No.** |  | **Primer sequences (5’ 🡪 3’)** |
| --- | --- | --- | --- | --- |
| 70 kDa heat shock protein, mitochondrial | *mthsp70/grp-75/mortalin* | DQ524993 | F | TCC GGT GTG GAT CTG ACC AAA GAC |
|  |  |  | R | TGT TTA GGC CCA GAA GCA TCC ATG |
| Apoptosis-related protein 1 | *aifm1* | JX975255 | F | ACA GAG GAG TCA GGA ACC |
|  |  |  | R | GGA GCA GGC AAT GAA GAG |
| Aryl hydrocarbon receptor 1 | *ahr1* | EU254480 | F | CCT GGG ACT GAA CGC CGA AG |
|  |  |  | R | GCT AAG TGT TGG GAT GTG GTT GG |
| ß-Actin | *actb* | X89920 | F | TCC TGC GGA ATC CAT GAG A |
|  |  |  | R | GAC GTC GCA CTT CAT GAT GCT |
| Carnitine palmitoyltransferase 1A | *cpt1a* | JQ308822 | F | GTG CCT TCG TTC GTT CCA TGA TC |
|  |  |  | R | TGA TGC TTA TCT GCT GCC TGT TTG |
| Catalase | *cat* | JQ308823 | F | TGG TCG AGA ACT TGA AGG CTG TC |
|  |  |  | R | AGG ACG CAG AAA TGG CAG AGG |
| Citrate synthase | *cs* | JX975229 | F | TCC AGG AGG TGA CGA GCC |
|  |  |  | R | GTG ACC AGC AGC CAG AAG AG |
| Cytochrome c oxidase assembly protein COX15 homolog | *cox15* | KC217651 | F | CAT ACT AGG TCG CTG GTT AG |
|  |  |  | R | GAT TCC GTG AGC CTT GTG |
| Cytochrome c oxidase subunit 4 isoform 1 | *cox4a* | JQ308835 | F | ACC CTG AGT CCA GAG CAG AAG TCC |
|  |  |  | R | AGC CAG TGA AGC CGA TGA GAA AGA AC |
| Cytochrome c oxidase subunit 5A, mitochondrial-like isoform 2 | *cox5a2* | KC217635 | F | CGC CAT CCG CAT CCT TGA |
|  |  |  | R | GGC TTC AAC TCT TGG ATC AGG TAG G |
| Cytochrome c oxidase subunit 6A isoform 2 | *cox6a2* | KC217639 | F | TGT TGG CTG CTG CGT CAC ATT C |
|  |  |  | R | CAG AAT CTT CCA GGT CCT CGC TCC |
| Cytochrome c oxidase subunit 6C1 | *cox6c1* | KC217642 | F | TCT CTC TGT CAC TCC TGG CTG CGA TAG |
|  |  |  | R | CCT GGG CTC TGT CAC TGC GTA CTT G |
| Cytochrome c oxidase subunit 7B | *cox7b* | KC217645 | F | TCT TCT GTG TGG CTG TGT GGT CAT ACG |
|  |  |  | R | TTC CCA ACA GGT GAC AAA TTC CAG GTG AT |
| Cytochrome c oxidase subunit 8B | *cox8b* | KC217648 | F | TCC GCT GGT CCC TGT GGC TAA |
|  |  |  | R | CCT CCA CTG ATA TTG TGT TTG GCA GGT TTG |
| Cytochrome c oxidase subunit I | *coxi* | KC217652 | F | GTC CTA CTT CTT CTG TCC CTT CCT GTT CT |
|  |  |  | R | AGG TTT CGG TCT GTA AGG AGC ATT GTA ATC |
| Cytochrome c oxidase subunit II | *coxii* | KC217653 | F | ACT GCC TAC ACA GGA CCT TGC C |
|  |  |  | R | GTC TGC TTC CAG GAG ACG GAA TTG T |
| Cytochrome c oxidase subunit III | *coxiii* | KC217654 | F | CCA AGC ACA CGC ATA CCA CAT A |
|  |  |  | R | GCG GCA ACT GCA CCT GTA |

**Suppl. Table 1.** (Continued)

| **Gene name** | **Symbol** | **Acc. No.** |  | **Primer sequences (5’ 🡪 3’)** |
| --- | --- | --- | --- | --- |
| Cytochrome P450 1A1 | *cyp1a1* | AF011223 | F | GCA TCA ACG ACC GCT TCA ACG C |
|  |  |  | R | CCT ACA ACC TTC TCA TCC GAC ATC TGG |
| Elongation of very long chain fatty acids 1 | *elovl1* | JX975700 | F | CTT CCT ACA CAT CTT CCA CCA CTC |
|  |  |  | R | CCA TTC CAC CAG GAG CAA AGG |
| Elongation of very long chain fatty acids 4 | *elovl4* | JX975701 | F | CGG TGG CAA TCA TCT TCC |
|  |  |  | R | TCA ACT GGC TGT CTG TGT |
| Elongation of very long chain fatty acids 5 | *elovl5* | AY660879 | F | CCT CCT GGT GCT CT ACA AT |
|  |  |  | R | GTG AGT GTC CTG GCA GTA |
| Elongation of very long chain fatty acids 6 | *elovl6* | JX975702 | F | GTG CTG CTC TAC TCC TGG TA |
|  |  |  | R | ACG GCA TGG ACC AAG TAG T |
| Estrogen receptor alpha | *er-α* | AF136979 | F | TCT AAG GGT CTG GAG CAC |
|  |  |  | R | TCG GTA TAG GGT CGG TTC |
| Fatty acid desaturase 2 | *fads2* | AY055749 | F | GCA GGC GGA GAG CGA CGG TCT GTT CC |
|  |  |  | R | AGC AGG ATG TGA CCC AGG TGG AGG CAG AAG |
| Follistatin | *fst* | AY544167 | F | GGA CCA GAC AAA CAA CGC ATA TTG |
|  |  |  | R | CAT AGA TGA TCC CGT CGT TTC CAC |
| Glucocorticoid receptor | *gcr* | DQ486890 | F | CCA GGA CAG GTG CCG AAC G |
|  |  |  | R | TGG AGG AAC TGC TGC TGA ACC |
| Glucose-regulated protein, 170 kDa | *grp-170* | JQ308821 | F | CAG AGG AGG CAG ACA GCA AGA C |
|  |  |  | R | TTC TCA GAC TCA GCA TTT CCA GAT TTC |
| Glucose-regulated protein, 94 kDa | *grp-94* | JQ308820 | F | AAG GCA CAG GCT TAC CAG ACA G |
|  |  |  | R | CTT CAG CAT CAT CGC CGA CTT TC |
| Glutathione peroxidase 4 | *gpx4* | AM977818 | F | TGC GTC TGA TAG GGT CCA CTG TC |
|  |  |  | R | GTC TGC CAG TCC TCT GTC GG |
| Glutathione reductase | *gr* | AJ937873 | F | TGT TCA GCC ACC CAC CCA TCG G |
|  |  |  | R | GCG TGA TAC ATC GGA GTG AAT GAA GTC TTG |
| Glutathione S-transferase 3 | *gst3* | JQ308828 | F | CCA GAT GAT CAG TAC GTG AAG ACC GTC |
|  |  |  | R | CTG CTG ATG TGA GGA ATG TAC CGT AAC |
| Growth hormone receptor I | *ghr-i* | AF438176 | F | ACCTGTCAGCCACCACATGA |
|  |  |  | R | TCGTGCAGATCTGGGTCGTA |
| Growth hormone receptor II | *ghr-ii* | AY573601 | F | GAGTGAACCCGGCCTGACAG |
|  |  |  | R | GCGGTGGTATCTGATTCATGGT |
| Hypoxia inducible factor-1 alpha | *hif-1α* | JQ308830 | F | CAG ATG AGC CTC TAA CTT GTG GAC |
|  |  |  | R | TTA GCA AGA ATG GTG GCA AGA TGA G |

**Suppl. Table 1.** (Continued 2)

| **Gene name** | **Symbol** | **Acc. No.** |  | **Primer sequences (5’ 🡪 3’)** |
| --- | --- | --- | --- | --- |
| Insulin receptor | *insr* | KM522774 | F | ACG GAC AGC AAG AAG GCA GAG AAT C |
|  |  |  | R | GGC TTC AAC GGT CGG ATC AGG T |
| Insulin-like growth factor binding protein 1a | *igfbp1a* | KM522771 | F | ACA AAC CAA AAC AGT GCG AGT CCT C |
|  |  |  | R | CCG TTC CAA GAG TTC ACA CAC CAG |
| Insulin-like growth factor binding protein 2b | *igfbp2b* | AF377998 | F | AGC GAT GTG TCC TGA GAT AGT GAG |
|  |  |  | R | GCA CCG TGG CGT GTA GAC C |
| Insulin-like growth factor binding protein 3 | *igfbp3** | MH577191  MH577192 | F | ACA GTG CCG TCC ATC CAA |
|  |  |  | R | GCT GCC CGT ATT TGT CCA |
| Insulin-like growth factor binding protein 4 | *igfbp4* | KM658998 | F | GGC ATC AAA CAC CCG CAC AC |
|  |  |  | R | ATC CAC GCA CCA GCA CTT CC |
| Insulin-like growth factor binding protein 5b | *igfbp5b* | MH577194 | F | CGA CAG GGC AGT CAA AGA AGC TAA CC |
|  |  |  | R | GTC TCG AAG GCA TGT GAG CAG AAG G |
| Insulin-like growth factor binding protein 6b | *igfbp6b* | MH577196 | F | GAT TGC TCA CTG CGG ATC |
|  |  |  | R | GGA GGG ACA GAC CTT GAA |
| Insulin-like growth factor receptor I | *igfr1* | KM522775 | F | TCA ACG ACA AGT ACG ACT ACC GCT GCT |
|  |  |  | R | CAC ACT TTC TGG CAC TGG TTG GAG GTC |
| Insulin-like growth factor receptor II | *igfr2* | KM522776 | F | ACA TTC GGG CAG CAC TCC TAA GAT |
|  |  |  | R | CCA GTT CAC CTC GTA GCG ACA GTT |
| Insulin-like growth factor-I | *igf-i* | AY996779 | F | TGTCTAGCGCTCTTTCCTTTCA |
|  |  |  | R | AGAGGGTGTGGCTACAGGAGATAC |
| Insulin-like growth factor-II | *igf-ii* | AY996778 | F | TGGGATCGTAGAGGAGTGTTGT |
|  |  |  | R | CTGTAGAGAGGTGGCCGACA |
| Lipoprotein lipase | *lpl* | AY495672 | F | CGT TGC CAA GTT TGT GAC CTG |
|  |  |  | R | AGG GTG TTC TGG TTG TCT GC |
| Mitochondrial fission factor homolog B | *miffb* | JX975252 | F | CGC AGC AGC ATT CCC TTC |
|  |  |  | R | CTC GTA CTG GAT TCG GTT CAT CT |
| Mitochondrial import inner membrane translocase subunit 23 | *tim23* | JX975240 | F | CAA GTC AGG AAG TGG CGT AA |
|  |  |  | R | AGA GCG TAG GCA CCA GAT A |
| Mitochondrial import inner membrane translocase subunit 44 | *tim44* | JX975239 | F | GAT GAC CTG GGA CAC ACT GG |
|  |  |  | R | TCA CTC CTC TTC CTG AGT CTG G |
| Mitochondrial import inner membrane translocase subunit Tim10 | *tim10* | JX975247 | F | TAC CGC CAC ATT ACA AGG AGC |
|  |  |  | R | ATC CAG GCA CAC CGA CTC |
| Mitochondrial import inner membrane translocase subunit Tim8A | *tim8a* | JX975245 | F | CGA CAC CAC CCT GAC CAT CAC |
|  |  |  | R | CGC CCT TCT GCA CCA TCT GT |

**Suppl. Table 1.** (Continued 3)

| **Gene name** | **Symbol** | **Acc. No.** |  | **Primer sequences (5’ 🡪 3’)** |
| --- | --- | --- | --- | --- |
| Mitochondrial import inner membrane translocase subunit Tim9 | *tim9* | JX975248 | F | CGT CAA AGA TTT CAC CAC CAG AGA G |
|  |  |  | R | GGA GAC ACG ACT CGG AGC A |
| Mitochondrial import receptor subunit Tom22 | *tom22* | JX975236 | F | CGC TCT GGG TGG GTA CTA CCT CCT T |
|  |  |  | R | CGA ACA CAA CAG GCA GCA CCA GGA T |
| Mitochondrial import receptor subunit Tom34 | *tom34* | JX975235 | F | GCT ACC GCC ACT TCT CCA CAA |
|  |  |  | R | TCT GTT TGG TGC CGT TCT GCT |
| Mitochondrial import receptor subunit Tom70 | *tom70* | JX975234 | F | GAG TCA GGT GGT CGA TAC A |
|  |  |  | R | CCA ATG AGC AGG TAG AAT GTG |
| Mitochondrial Rho GTPase 1 | *aifm1* | JX975255 | F | ACA GAG GAG TCA GGA ACC |
|  |  |  | R | GGA GCA GGC AAT GAA GAG |
| Mitochondrial Rho GTPase 2 | *aifm1* | JX975255 | F | ACA GAG GAG TCA GGA ACC |
|  |  |  | R | GGA GCA GGC AAT GAA GAG |
| Mitofusin 2 | *mfn2* | JX975251 | F | GGG ATG CCT CAG CCT CAG AAC CT |
|  |  |  | R | CTG CCT GCG GAC CTC TTC CAT GTA TT |
| Myoblast determination protein 1 | *myod1* | AF478568 | F | ATG GAG CTG TCG GAT ATC TCT TTC |
|  |  |  | R | GAA GCA GGG GTC ATC GTA GAA ATC |
| Myocyte-specific enhancer factor 2A | *mef2a* | KM522777 | F | ATG GAC GAG AGG AAC AGG CAG GTT A |
|  |  |  | R | GGC TAT CTC ACA GTC ACA TAG TAC GCT CAG |
| Myocyte-specific enhancer factor 2C | *mef2c* | KM522778 | F | TAG CAA CTC CCA CTC TAC CAG GAC AAG |
|  |  |  | R | GGA ATA CTC GGC ACC ATA AGA AGT CG |
| Myogenic factor 5 | *myf5* | JN034420 | F | GCA TGG TTG ACA GCA ACA GTC CAG TGT |
|  |  |  | R | TGT CTT ATC GCC CAA AGT GTC GTT CTT CAT |
| Myogenic factor 6 | *myf6/mrf4/ herculin* | JN034421 | F | GCA GCA ATG ACA AAC CAG AGA GAC GGA ACA |
|  |  |  | R | GAG GCT GGA GGA CGC CGA AGA TTC A |
| Myogenic factor MYOD2 | *myod2* | AF478569 | F | CCA ACT GCT CTG ATG GCA TGA TGG ATT TC |
|  |  |  | R | GAC CGT TTG CTT CTC CTG GAC TCG TAT G |
| Myostatin/Growth differentiation factor 8 | *mstn/gdf-8* | AF258448 | F | AAG AGC AGA TCA TCT ACG GCA AGA TCC |
|  |  |  | R | TCA AGA GCA TCC ACA ACG GTC TAC CA |
| NADH dehydrogenase [ubiquinone] 1 alpha subcomplex, assembly factor 2 | *ndufaf2* | KC217598 | F | AGG CAG CAT ACC GAT AGA G |
|  |  |  | R | ACT CAT TCT TCA GCA ACT CCT |
| NADH dehydrogenase [ubiquinone] 1 alpha subcomplex subunit 1 | *ndufa1* | KC217562 | F | CGG GTT CCG TGG CAG TGG TA |
|  |  |  | R | TCC TGT TCC TGA TAC TCG CTT GTC TCT |

**Suppl. Table 1.** (Continued 4)

| **Gene name** | **Symbol** | **Acc. No.** |  | **Primer sequences (5’ 🡪 3’)** |
| --- | --- | --- | --- | --- |
| NADH dehydrogenase [ubiquinone] 1 alpha subcomplex subunit 3 | *ndufa3* | KC217564 | F | TCG GAG CGT TCC TGA AGA ATG C |
|  |  |  | R | GAA GAG CCA TAC CTA TCA GTC CAA TAC CA |
| NADH dehydrogenase [ubiquinone] 1 alpha subcomplex subunit 4 | *ndufa4* | KC217565 | F | GCT CGT CTG GGC TTG AGA AAC C |
|  |  |  | R | GCT CTG GGT TGT TCT TGC GAT CC |
| NADH dehydrogenase [ubiquinone] 1 alpha subcomplex subunit 7 | *ndufa7* | KC217569 | F | CCG AGC CAC AAG TAT GCC AGC AAC TA |
|  |  |  | R | AGC CTC CCT GCG TCC ATC TCT G |
| NADH dehydrogenase [ubiquinone] 1 beta subcomplex subunit 5 | *ndufb5* | KC217580 | F | TGC GTC GGC AGA TGA GGA T |
|  |  |  | R | CTT GTT GAG GGT GTT CAC CTG GAA |
| NADH dehydrogenase iron-sulfur protein 2 | *ndufs2* | KC217589 | F | GTA TCA GAC GGC TCC AGC AGA C |
|  |  |  | R | AGA CCA GCC AAG TGA GCG AAT |
| NADH dehydrogenase iron-sulfur protein 7 | *ndufs7* | KC217594 | F | AAC GGA GGA GGC TAC TAC CAC TAC T |
|  |  |  | R | CGG TAC GAT TCG GTC ACA ACC TCT AAC |
| NADH-ubiquinone oxidoreductase chain 2 | *nd2* | KC217558 | F | TAG GTT GAA TGA CCA TCG TA |
|  |  |  | R | GGC TAA GGA GTT GAG GTT |
| NADH-ubiquinone oxidoreductase chain 5 | *nd5* | KC217559 | F | CCT AAA CGC CTG AGC CCT GG |
|  |  |  | R | GCT GTA AAC GAG GTG GCT AGA AGG |
| Nuclear respiratory factor 1 | *nrf1* | JX975263 | F | CAG ATA GTC CTG GCA GAG A |
|  |  |  | R | GAC CTG TGG CAT CTT GAA |
| Peroxiredoxin 3 | *prdx3* | GQ252681 | F | ATC AAC ACC CCA CGC AAG ACT G |
|  |  |  | R | ACC GTT TGG ATC AAT GAG GAA CAG ACC |
| Peroxiredoxin 5 | *prdx5* | GQ252683 | F | ATC AAC ACC CCA CGC AAG ACT G |
|  |  |  | R | TCC ACA TTG ATC TTC TTC ACG ACT CC |
| Peroxisome proliferator-activated receptor α | *pparα* | AY590299 | F | TCT CTT CAG CCC ACC ATC CC |
|  |  |  | R | ATC CCA GCG TGT CGT CTC C |
| Peroxisomeproliferator-activated receptor γ | *pparγ* | AY590304 | F | CGC CGT GGA CCT GTC AGA GC |
|  |  |  | R | GGA ATG GAT GGA GGA GGA GGA GAT GG |
| Proliferator-activated receptor gamma coactivator 1 alpha | *pgc1α* | JX975264 | F | CGT GGG ACA GGT GTA ACC AGG ACT C |
|  |  |  | R | ACC AAC CAA GGC AGC ACA CTC TAA TTC T |
| Proliferator-activated receptor gamma coactivator 1 beta | *pgc1β* | JX975265 | F | TCA GAG GAA GAG GCG GAT |
|  |  |  | R | GAC ACA GGT GGA GGA TGG |
| SCO1 protein homolog, mitochondrial | *sco1* | KC217649 | F | ACA ACA ACA AGC CCA CCA AGA |
|  |  |  | R | GAC AGT GAG TGA ACC CGA AGT AGA T |

**Suppl. Table 1.** (Continued 5)

| **Gene name** | **Symbol** | **Acc. No.** |  | **Primer sequences (5’ 🡪 3’)** |
| --- | --- | --- | --- | --- |
| Sirtuin1 | *sirt1* | KF018666 | F | GGT TCC TAC AGT TTC ATC CAG CAG CAC ATC |
|  |  |  | R | CCT CAG AAT GGT CCT CGG ATC GGT CTC |
| Sirtuin2 | *sirt2* | KF018667 | F | GAA CAA TCC GAC GAC AGC AGT GAA G |
|  |  |  | R | AGG TTA CGC AGG AAG TCC ATC TCT |
| Sirtuin3 | *sirt3* | KF018668 | F | CTG CCA AGT CCT CAT CCC |
|  |  |  | R | CTT CAC CAG ACG AGC CAC |
| Sirtuin4 | *sirt4* | KF018669 | F | GGC TGG CGG AGT CGG ATG |
|  |  |  | R | TCC TGA ATA CAC CTG TGA CGA AGA C |
| Sirtuin5 | *sirt5* | KF018670 | F | CAG ACA TCC TAA CCC GAG CAG AG |
|  |  |  | R | CCA CGA GGC AGA GGT CAC A |
| Sirtuin6 | *sirt6* | KF018671 | F | ACT CCA CCA CCA CCG ATG TCA A |
|  |  |  | R | CTC CTC CTC CTT CAC CTT TCG CTT TG |
| Sirtuin7 | *sirt7* | KF018672 | F | CTG GAG CAA CCT CTA AAC TGG AA |
|  |  |  | R | CAC CTT CAG ACT GGA GCC TAA |
| Stearoyl-CoA desaturase 1a | *scd1a* | JQ277703 | F | CGG AGG CGG AGG CGT TGG AGA AGA AG |
|  |  |  | R | AGG GAG ACG GCG TAC AGG GCA CCT ATA TG |
| Stearoyl-CoA desaturase 1b | *scd1b* | JQ277704 | F | GCT CAA TCT CAC CAC CGC CTT CAT AG |
|  |  |  | R | GCT GCC GTC GCC CGT TCT CTG |
| Superoxide dismutase [Mn] | *Mn-sod/sod2* | JQ308833 | F | CCT GAC CTG ACC TAC GAC TAT GG |
|  |  |  | R | AGT GCC TCC TGA TAT TTC TCC TCT G |
| Surfeit locus protein 1 | *surf1* | KC217650 | F | AGA TGG AAG GTG AAG TGG AGG TGG TC |
|  |  |  | R | GCG TTG CTC TGT CTG CCG AAC T |
| Uncoupling protein 1 | *ucp1* | FJ710211 | F | GCA CAC TAC CCA ACA TCA CAA G |
|  |  |  | R | CGC CGA ACG CAG AAA CAA AG |
| Uncoupling protein 2 | *ucp2* | JQ859959 | F | CGG CGG CGT CCT CAG TTG |
|  |  |  | R | AAG CAA GTG GTC CCT CTT TGG TCA T |
| Uncoupling protein 3 | *ucp3* | EU555336 | F | AGG TGC GAC TGG CTG ACG |
|  |  |  | R | TTC GGC ATA CAA CCT CTC CAA AG |

(*) Acc. No. MH577191: *igfbp3a*; Acc. No. MH577192: *igfbp3b*. Primers used for *igfbp3* gene expression jointly amplify both *igfbp3a* and *igfbp3b* isoforms.
