# Supplementary material for: Tissue-Specific Orchestration of Gilthead Sea Bream Resilience to Hypoxia and High Stocking Density
Source: Front Physiol. 2019 Jul 10;10:840. doi: 10.3389/fphys.2019.00840 (PMC6635561; doi:10.3389/fphys.2019.00840)
Supplement: Supplementary file 4 [file Table_2.docx]

**Suppl. Table 2.** Effects of rearing density and dissolved oxygen level on gilthead sea bream relative expression of hepatic selected genes on a 3-week feeding trial. Values on relative expression are the mean ± SEM of 8 fish (2-3 fish per replicate tank). P-values are the result of two-way analysis of variance. Non-significance (P>0.05) is stated by “n.s”. Asterisks in each row indicate significant differences with oxygen level for a given rearing density (SNK test, P<0.05).

| Category | Symbol | LD | |  | HD | |  | P-value | | |
| --- | --- | --- | --- | --- | --- | --- | --- | --- | --- | --- |
|  |  | Normoxia | Hypoxia |  | Normoxia | Hypoxia |  | [O_2_] | Density | Interaction |
| GH/IGF system | *ghr-i* | 3.06 ± 0.27 | 2.18 ± 0.26* |  | 3.70 ± 0.36 | 2.79 ± 0.35* |  | 0.007 | 0.049 | n.s. |
|  | *ghr-ii* | 2.39 ± 0.25 | 2.62 ± 0.35 |  | 4.00 ± 0.59 | 2.70 ± 0.26 |  | n.s. | 0.032 | n.s. |
|  | *igf-i* | 9.51 ± 0.58 | 9.26 ± 1.20 |  | 13.29 ± 1.86 | 11.94 ± 0.79 |  | n.s. | 0.013 | n.s. |
|  | *igf-ii* | 2.85 ± 0.44 | 3.73 ± 0.53 |  | 5.37 ± 0.57 | 3.31 ± 0.76* |  | n.s. | n.s. | 0.022 |
|  | *igfbp1a* | 0.03 ± 0.00 | 0.03 ± 0.00 |  | 0.03 ± 0.00 | 0.04 ± 0.00 |  | n.s. | n.s. | n.s. |
|  | *igfbp2b* | 3.17 ± 0.22 | 3.29 ± 0.22 |  | 3.64 ± 0.40 | 3.11 ± 0.17 |  | n.s. | n.s. | n.s. |
|  | *igfbp4* | 0.81 ± 0.09 | 0.79 ± 0.10 |  | 0.82 ± 0.13 | 0.85 ± 0.06 |  | n.s. | n.s. | n.s. |
| Lipid metabolism | *elovl1* | 10.63 ± 0.46 | 13.41 ± 0.79* |  | 10.53 ± 0.66 | 12.69 ± 1.01 |  | 0.003 | n.s. | n.s. |
|  | *elovl4* | 0.44 ± 0.05 | 0.49 ± 0.03 |  | 0.52 ± 0.05 | 0.41 ± 0.03 |  | n.s. | n.s. | n.s. |
|  | *elovl5* | 2.67 ± 0.55 | 3.32 ± 0.65 |  | 4.14 ± 1.11 | 2.17 ± 0.25 |  | n.s. | n.s. | n.s. |
|  | *elovl6* | 0.89 ± 0.12 | 0.89 ± 0.14 |  | 0.52 ± 0.06 | 0.60 ± 0.07 |  | n.s. | 0.004 | n.s. |
|  | *fads2* | 3.82 ± 0.42 | 9.19 ± 0.97*** |  | 5.10 ± 0.60 | 4.98 ± 0.66 |  | <0.001 | 0.048 | <0.001 |
|  | *scd1a* | 0.22 ± 0.03 | 0.52 ± 0.13* |  | 0.22 ± 0.05 | 0.19 ± 0.02 |  | n.s. | 0.024 | 0.026 |
|  | *scd1b* | 0.43 ± 0.09 | 1.15 ± 0.19** |  | 0.53 ± 0.23 | 0.56 ± 0.14 |  | 0.042 | 0.018 | 0.050 |
|  | *lpl* | 5.77 ± 0.78 | 6.57 ± 0.56 |  | 8.08 ± 0.99 | 8.75 ± 0.82 |  | n.s. | 0.010 | n.s. |
|  | *pparα* | 2.26 ± 0.26 | 2.69 ± 0.20 |  | 2.29 ± 0.28 | 2.84 ± 0.24 |  | n.s. | n.s. | n.s. |
|  | *pparγ* | 0.78 ± 0.06 | 0.83 ± 0.09 |  | 0.87 ± 0.09 | 0.71 ± 0.04 |  | n.s. | n.s. | n.s. |
| Energy sensing and oxidative metabolism | *sirt1* | 0.10 ± 0.01 | 0.09 ± 0.01 |  | 0.12 ± 0.01 | 0.10 ± 0.01 |  | n.s. | n.s. | n.s. |
|  | *sirt2* | 0.30 ± 0.01 | 0.31 ± 0.02 |  | 0.34 ± 0.03 | 0.28 ± 0.01 |  | n.s. | n.s. | n.s. |
|  | *sirt3* | 0.05 ± 0.00 | 0.05 ± 0.00 |  | 0.05 ± 0.00 | 0.04 ± 0.00 |  | n.s. | n.s. | n.s. |
|  | *sirt4* | 0.02 ± 0.00 | 0.02 ± 0.00 |  | 0.02 ± 0.00 | 0.02 ± 0.00 |  | n.s. | n.s. | n.s. |
|  | *sirt5* | 0.32 ± 0.02 | 0.33 ± 0.02 |  | 0.33 ± 0.03 | 0.30 ± 0.02 |  | n.s. | n.s. | n.s. |
|  | *sirt6* | 0.03 ± 0.00 | 0.04 ± 0.00 |  | 0.04 ± 0.00 | 0.03 ± 0.00 |  | n.s. | n.s. | n.s. |
|  | *sirt7* | 0.05 ± 0.00 | 0.05 ± 0.00 |  | 0.05 ± 0.00 | 0.05 ± 0.00 |  | n.s. | n.s. | n.s. |
|  | *cpt1a* | 0.71 ± 0.11 | 0.50 ± 0.04 |  | 0.62 ± 0.10 | 0.59 ± 0.06 |  | n.s. | n.s. | n.s. |
|  | *cs* | 1.02 ± 0.08 | 0.93 ± 0.05 |  | 0.98 ± 0.08 | 0.93 ± 0.07 |  | n.s. | n.s. | n.s. |
|  | *nd2* | 37.29 ± 2.44 | 29.65 ± 2.15* |  | 38.21 ± 3.14 | 26.85 ± 2.26* |  | <0.001 | n.s. | n.s. |
|  | *nd5* | 16.51 ± 0.83 | 14.41 ± 0.73 |  | 18.11 ± 1.54 | 14.20 ± 1.11* |  | 0.010 | n.s. | n.s. |
|  | *coxi* | 65.74 ± 4.70 | 71.34 ± 7.10 |  | 76.95 ± 7.56 | 69.25 ± 3.24 |  | n.s. | n.s. | n.s. |
|  | *coxii* | 44.07 ± 2.34 | 37.08 ± 1.90* |  | 39.36 ± 3.29 | 39.94 ± 2.30 |  | n.s. | n.s. | n.s. |
|  | *ucp1* | 23.04 ± 2.14 | 19.01 ± 1.99 |  | 17.55 ± 2.17 | 15.98 ± 1.46 |  | n.s. | 0.002 | n.s. |
|  | *pgc1α* | 0.06 ± 0.01 | 0.07 ± 0.01 |  | 0.10 ± 0.02 | 0.12 ± 0.02 |  | n.s. | 0.010 | n.s. |
|  | *pgc1β* | 0.42 ± 0.06 | 0.50 ± 0.07 |  | 0.71 ± 0.08 | 0.39 ± 0.06** |  | n.s. | n.s. | 0.008 |
|  | *hif-1α* | 1.12 ± 0.07 | 1.14 ± 0.09 |  | 1.24 ± 0.13 | 1.05 ± 0.04 |  | n.s. | n.s. | n.s. |
| Antioxidant defence and  tissue repair | *cat* | 23.55 ± 1.75 | 22.09 ± 2.76 |  | 21.97 ± 1.98 | 21.21 ± 1.10 |  | n.s. | n.s. | n.s. |
|  | *gpx4* | 21.34 ± 2.51 | 14.41 ± 1.48* |  | 18.20 ± 2.14 | 12.28 ± 1.03* |  | 0.002 | n.s. | n.s. |
|  | *gr* | 0.36 ± 0.02 | 0.34 ± 0.02 |  | 0.42 ± 0.04 | 0.28 ± 0.02** |  | 0.006 | n.s. | 0.050 |
|  | *prdx3* | 0.82 ± 0.05 | 0.87 ± 0.05 |  | 0.94 ± 0.07 | 0.72 ± 0.07* |  | n.s. | n.s. | 0.030 |
|  | *prdx5* | 0.59 ± 0.03 | 0.44 ± 0.03** |  | 0.60 ± 0.06 | 0.43 ± 0.02* |  | <0.001 | n.s. | n.s. |
|  | *Mn-sod / sod2* | 1.20 ± 0.05 | 1.02 ± 0.08 |  | 1.30 ± 0.09 | 0.94 ± 0.08* |  | 0.001 | n.s. | n.s. |
|  | *grp-170* | 1.03 ± 0.08 | 1.41 ± 0.17 |  | 1.37 ± 0.19 | 1.11 ± 0.08 |  | n.s. | n.s. | 0.023 |
|  | *grp-94* | 3.44 ± 0.40 | 3.90 ± 0.48 |  | 3.37 ± 0.46 | 3.60 ± 0.42 |  | n.s. | n.s. | n.s. |
|  | *mthsp70/grp-75/mortalin* | 0.56 ± 0.04 | 0.55 ± 0.04 |  | 0.80 ± 0.10 | 0.55 ± 0.05* |  | 0.033 | 0.044 | n.s. |
