# Supplementary material for: Tissue-Specific Orchestration of Gilthead Sea Bream Resilience to Hypoxia and High Stocking Density
Source: Front Physiol. 2019 Jul 10;10:840. doi: 10.3389/fphys.2019.00840 (PMC6635561; doi:10.3389/fphys.2019.00840)
Supplement: Supplementary file 5 [file Table_3.docx]

**Suppl. Table 3.** Effects of rearing density and dissolved oxygen level on gilthead sea bream relative expression of white muscle selected genes on a 3-week feeding trial. Values on relative expression are the mean ± SEM of 8 fish (2-3 fish per replicate tank). P-values are the result of two-way analysis of variance. Non-significance (P>0.05) is stated by “n.s”. Asterisks in each row indicate significant differences with oxygen level for a given rearing density (SNK test, P<0.05).

| Category | Symbol | LD | |  | HD | |  | P-value | | |
| --- | --- | --- | --- | --- | --- | --- | --- | --- | --- | --- |
|  |  | Normoxia | Hypoxia |  | Normoxia | Hypoxia |  | [O_2_] | Density | Interaction |
| GH/IGF system | *ghr-i* | 6.42 ± 0.57 | 5.63 ± 0.20 |  | 4.14 ± 0.40 | 4.84 ± 0.51 |  | n.s. | 0.002 | n.s. |
|  | *ghr-ii* | 2.71 ± 0.41 | 3.32 ± 0.50 |  | 2.83 ± 0.42 | 3.44 ± 0.71 |  | n.s. | n.s. | n.s. |
|  | *igf-i* | 0.76 ± 0.09 | 0.67 ± 0.06 |  | 0.65 ± 0.11 | 0.63 ± 0.08 |  | n.s. | n.s. | n.s. |
|  | *igf-ii* | 3.16 ± 0.28 | 3.50 ± 0.28 |  | 2.49 ± 0.27 | 3.03 ± 0.25 |  | n.s. | 0.043 | n.s. |
|  | *igfbp3* | 9.33 ± 0.89 | 11.68 ± 1.03 |  | 13.17 ± 0.99 | 14.97 ± 2.18 |  | n.s. | 0.015 | n.s. |
|  | *igfbp5b* | 5.16 ± 0.39 | 6.21 ± 0.35 |  | 7.08 ± 0.47 | 6.18 ± 0.29 |  | n.s. | 0.019 | 0.016 |
|  | *igfbp6b* | 0.53 ± 0.05 | 0.59 ± 0.06 |  | 1.01 ± 0.10 | 0.65 ± 0.05* |  | n.s. | 0.001 | 0.009 |
|  | *insr* | 3.17 ± 0.15 | 3.26 ± 0.29 |  | 4.30 ± 0.43 | 3.28 ± 0.17* |  | n.s. | 0.050 | n.s. |
|  | *igfr1* | 1.76 ± 0.09 | 1.81 ± 0.09 |  | 2.36 ± 0.21 | 1.93 ± 0.12 |  | n.s. | 0.014 | n.s. |
|  | *igfr2* | 0.93 ± 0.08 | 0.89 ± 0.06 |  | 1.15 ± 0.08 | 0.81 ± 0.03* |  | 0.005 | n.s. | 0.029 |
| Muscle growth and cell differentiation | *myod1* | 27.87 ± 2.12 | 25.73 ± 1.72 |  | 31.73 ± 2.85 | 29.29 ± 2.00 |  | n.s. | n.s. | n.s. |
|  | *myod2* | 10.96 ± 0.82 | 12.83 ± 0.61 |  | 10.78 ± 0.63 | 13.29 ± 1.03 |  | 0.010 | n.s. | n.s. |
|  | *myf5* | 1.41 ± 0.09 | 1.41 ± 0.11 |  | 1.32 ± 0.11 | 1.63 ± 0.14 |  | n.s. | n.s. | n.s. |
|  | *myf6/mrf4* | 0.85 ± 0.05 | 0.85 ± 0.04 |  | 0.95 ± 0.06 | 0.92 ± 0.04 |  | n.s. | n.s. | n.s. |
|  | *mstn/gdf-8* | 11.95 ± 1.31 | 13.93 ± 1.95 |  | 9.89 ± 0.36 | 12.45 ± 1.54 |  | n.s. | n.s. | n.s. |
|  | *mef2a* | 53.53 ± 4.03 | 57.79 ± 5.17 |  | 59.61 ± 3.72 | 57.52 ± 2.69 |  | n.s. | n.s. | n.s. |
|  | *mef2c* | 14.88 ± 0.89 | 13.95 ± 0.66 |  | 15.38 ± 0.91 | 14.87 ± 0.74 |  | n.s. | n.s. | n.s. |
|  | *fst* | 1.63 ± 0.06 | 1.66 ± 0.19 |  | 1.39 ± 0.18 | 1.69 ± 0.16 |  | n.s. | n.s. | n.s. |
| Energy sensing and oxidative metabolism | *sirt1* | 0.73 ± 0.04 | 0.70 ± 0.04 |  | 0.84 ± 0.04 | 0.72 ± 0.03* |  | 0.050 | n.s. | n.s. |
|  | *sirt2* | 1.45 ± 0.06 | 1.49 ± 0.08 |  | 1.61 ± 0.14 | 1.43 ± 0.06 |  | n.s. | n.s. | n.s. |
|  | *sirt3* | 0.19 ± 0.01 | 0.19 ± 0.02 |  | 0.20 ± 0.02 | 0.20 ± 0.01 |  | n.s. | n.s. | n.s. |
|  | *sirt4* | 0.15 ± 0.01 | 0.14 ± 0.01 |  | 0.17 ± 0.02 | 0.18 ± 0.01 |  | n.s. | 0.025 | n.s. |
|  | *sirt5* | 2.15 ± 0.22 | 2.23 ± 0.17 |  | 2.28 ± 0.18 | 2.34 ± 0.16 |  | n.s. | n.s. | n.s. |
|  | *sirt6* | 0.16 ± 0.01 | 0.14 ± 0.01 |  | 0.15 ± 0.01 | 0.17 ± 0.02 |  | n.s. | n.s. | n.s. |
|  | *sirt7* | 0.24 ± 0.01 | 0.25 ± 0.02 |  | 0.27 ± 0.01 | 0.28 ± 0.02 |  | n.s. | n.s. | n.s. |
|  | *cpt1a* | 9.05 ± 1.02 | 6.72 ± 0.43* |  | 7.44 ± 0.46 | 10.74 ± 1.25* |  | n.s. | n.s. | 0.002 |
|  | *cs* | 53.95 ± 3.69 | 50.43 ± 3.17 |  | 53.22 ± 4.00 | 55.01 ± 2.65 |  | n.s. | n.s. | n.s. |
|  | *nd2* | 251.51 ± 20.79 | 217.53 ± 15.18 |  | 250.45 ± 25.13 | 231.71 ± 20.24 |  | n.s. | n.s. | n.s. |
|  | *nd5* | 151.75 ± 13.47 | 125.16 ± 6.09 |  | 135.62 ± 8.33 | 123.75 ± 10.63 |  | n.s. | n.s. | n.s. |
|  | *coxi* | 740.24 ± 58.36 | 732.57 ± 26.90 |  | 834.22 ± 61.65 | 957.89 ± 57.10 |  | n.s. | 0.005 | n.s. |
|  | *coxii* | 471.08 ± 49.13 | 453.37 ± 26.52 |  | 496.06 ± 57.85 | 474.56 ± 27.28 |  | n.s. | n.s. | n.s. |
|  | *ucp3* | 11.65 ± 1.78 | 6.43 ± 0.93* |  | 16.89 ± 4.46 | 6.91 ± 0.74* |  | 0.006 | n.s. | n.s. |
|  | *pgc1α* | 0.67 ± 0.21 | 0.30 ± 0.09 |  | 0.35 ± 0.07 | 0.27 ± 0.07 |  | n.s. | n.s. | n.s. |
|  | *pgc1β* | 1.30 ± 0.18 | 1.01 ± 0.06 |  | 1.12 ± 0.14 | 1.15 ± 0.09 |  | n.s. | n.s. | n.s. |
|  | *hif-1α* | 5.45 ± 0.35 | 4.55 ± 0.30 |  | 6.67 ± 0.69 | 5.51 ± 0.31 |  | 0.028 | 0.020 | n.s. |
| Antioxidant defence and  tissue repair | *cat* | 9.19 ± 0.61 | 7.64 ± 0.46 |  | 9.19 ± 0.80 | 9.17 ± 0.49 |  | n.s. | n.s. | n.s. |
|  | *gpx4* | 1.78 ± 0.48 | 1.01 ± 0.39 |  | 0.78 ± 0.21 | 0.43 ± 0.08 |  | n.s. | 0.038 | n.s. |
|  | *gr* | 0.99 ± 0.05 | 0.88 ± 0.07 |  | 1.08 ± 0.08 | 0.98 ± 0.06 |  | n.s. | n.s. | n.s. |
|  | *prdx3* | 10.44 ± 0.97 | 9.60 ± 0.45 |  | 9.96 ± 0.77 | 9.42 ± 0.94 |  | n.s. | n.s. | n.s. |
|  | *prdx5* | 28.69 ± 1.98 | 20.68 ± 1.10** |  | 27.18 ± 2.14 | 22.48 ± 1.14* |  | <0.001 | n.s. | n.s. |
|  | *Mn-sod / sod2* | 9.81 ± 0.68 | 8.31 ± 0.33 |  | 11.16 ± 1.25 | 8.92 ± 0.46 |  | 0.021 | n.s. | n.s. |
|  | *grp-170* | 1.80 ± 0.08 | 1.93 ± 0.12 |  | 2.09 ± 0.12 | 1.72 ± 0.11* |  | n.s. | n.s. | 0.025 |
|  | *grp-94* | 4.28 ± 0.28 | 4.70 ± 0.35 |  | 4.76 ± 0.40 | 4.29 ± 0.31 |  | n.s. | n.s. | n.s. |
|  | *mthsp70/grp-75/mortalin* | 5.58 ± 0.27 | 5.86 ± 0.43 |  | 6.96 ± 0.65 | 5.83 ± 0.47 |  | n.s. | n.s. | n.s. |
