# Supplementary material for: Tissue-Specific Orchestration of Gilthead Sea Bream Resilience to Hypoxia and High Stocking Density
Source: Front Physiol. 2019 Jul 10;10:840. doi: 10.3389/fphys.2019.00840 (PMC6635561; doi:10.3389/fphys.2019.00840)
Supplement: Supplementary file 6 [file Table_4.docx]

**Suppl. Table 4.** Effects of rearing density and dissolved oxygen level on gilthead sea bream relative expression of cardiac selected genes on a 3-week feeding trial. Values on relative expression are the mean ± SEM of 8 fish (2-3 fish per replicate tank). P-values are the result of two-way analysis of variance. Non-significance (P>0.05) is stated by “n.s”. Asterisks in each row indicate significant differences with oxygen level for a given rearing density (SNK test, P<0.05).

| Category | Symbol | LD | |  | HD | |  | P-value | | |
| --- | --- | --- | --- | --- | --- | --- | --- | --- | --- | --- |
|  |  | Normoxia | Hypoxia |  | Normoxia | Hypoxia |  | [O_2_] | Density | Interaction |
| GH/IGF system | *ghr-i* | 0.39 ± 0.01 | 0.35 ± 0.02 |  | 0.38 ± 0.02 | 0.30 ± 0.03 |  | 0.021 | n.s. | n.s. |
|  | *ghr-ii* | 1.85 ± 0.13 | 1.77 ± 0.14 |  | 2.11 ± 0.08 | 1.73 ± 0.21 |  | n.s. | n.s. | n.s. |
|  | *igf-i* | 0.01 ± 0.00 | 0.01 ± 0.00 |  | 0.01 ± 0.00 | 0.01 ± 0.00 |  | n.s. | n.s. | n.s. |
|  | *igf-ii* | 2.22 ± 0.21 | 2.26 ± 0.14 |  | 2.51 ± 0.17 | 1.88 ± 0.24* |  | n.s. | n.s. | n.s. |
| Energy sensing and oxidative metabolism | *sirt1* | 0.22 ± 0.01 | 0.20 ± 0.01 |  | 0.21 ± 0.01 | 0.18 ± 0.01* |  | 0.023 | n.s. | n.s. |
|  | *sirt2* | 0.34 ± 0.02 | 0.31 ± 0.02 |  | 0.33 ± 0.03 | 0.28 ± 0.01 |  | n.s. | n.s. | n.s. |
|  | *sirt3* | 0.10 ± 0.00 | 0.11 ± 0.01 |  | 0.09 ± 0.00 | 0.09 ± 0.00 |  | n.s. | 0.036 | n.s. |
|  | *sirt4* | 0.06 ± 0.00 | 0.05 ± 0.00 |  | 0.05 ± 0.00 | 0.05 ± 0.00 |  | n.s. | n.s. | n.s. |
|  | *sirt5* | 0.99 ± 0.08 | 0.94 ± 0.05 |  | 0.96 ± 0.05 | 0.75 ± 0.04** |  | 0.023 | n.s. | n.s. |
|  | *sirt6* | 0.05 ± 0.00 | 0.05 ± 0.00 |  | 0.05 ± 0.00 | 0.04 ± 0.00* |  | 0.042 | n.s. | n.s. |
|  | *sirt7* | 0.07 ± 0.01 | 0.06 ± 0.00 |  | 0.07 ± 0.01 | 0.05 ± 0.00* |  | 0.003 | n.s. | n.s. |
|  | *cpt1a* | 1.63 ± 0.15 | 1.28 ± 0.12 |  | 1.23 ± 0.12 | 1.21 ± 0.10 |  | n.s. | n.s. | n.s. |
|  | *cs* | 9.29 ± 0.54 | 7.79 ± 0.47* |  | 7.95 ± 0.25 | 6.80 ± 0.25** |  | 0.002 | 0.007 | n.s. |
|  | *nd2* | 68.14 ± 4.68 | 70.41 ± 5.69 |  | 64.19 ± 5.02 | 52.61 ± 2.44* |  | n.s. | 0.026 | n.s. |
|  | *nd5* | 45.09 ± 3.28 | 40.14 ± 2.49 |  | 44.19 ± 3.70 | 31.03 ± 0.90** |  | 0.003 | n.s. | n.s. |
|  | *coxi* | 329.33 ± 18.41 | 310.66 ± 14.83 |  | 332.42 ± 27.46 | 278.19 ± 19.29 |  | n.s. | n.s. | n.s. |
|  | *coxii* | 84.97 ± 5.14 | 99.58 ± 8.00 |  | 83.61 ± 5.07 | 80.59 ± 5.62 |  | n.s. | n.s. | n.s. |
|  | *ucp2* | 1.88 ± 0.23 | 1.78 ± 0.18 |  | 1.97 ± 0.28 | 1.62 ± 0.15 |  | n.s. | n.s. | n.s. |
|  | *pgc1α* | 2.55 ± 0.15 | 2.28 ± 0.15 |  | 2.74 ± 0.26 | 2.08 ± 0.10* |  | 0.012 | n.s. | n.s. |
|  | *pgc1β* | 0.97 ± 0.04 | 0.85 ± 0.06 |  | 0.96 ± 0.05 | 0.84 ± 0.05 |  | 0.026 | n.s. | n.s. |
|  | *hif-1α* | 6.67 ± 0.57 | 5.35 ± 0.36* |  | 6.15 ± 0.31 | 4.52 ± 0.28* |  | 0.001 | n.s. | n.s. |
| Antioxidant defence and  tissue repair | *cat* | 2.06 ± 0.16 | 1.59 ± 0.10* |  | 1.73 ± 0.10 | 1.56 ± 0.10 |  | 0.001 | n.s. | n.s. |
|  | *gpx4* | 0.09 ± 0.01 | 0.10 ± 0.03 |  | 0.10 ± 0.01 | 0.09 ± 0.01 |  | n.s. | n.s. | n.s. |
|  | *gr* | 0.42 ± 0.03 | 0.40 ± 0.03 |  | 0.37 ± 0.01 | 0.33 ± 0.01* |  | n.s. | 0.012 | n.s. |
|  | *prdx3* | 2.14 ± 0.15 | 2.08 ± 0.17 |  | 1.97 ± 0.13 | 1.61 ± 0.14 |  | n.s. | 0.041 | n.s. |
|  | *prdx5* | 6.26 ± 0.52 | 4.34 ± 0.40* |  | 4.95 ± 0.30 | 3.95 ± 0.16* |  | <0.001 | 0.029 | n.s. |
|  | *Mn-sod / sod2* | 2.52 ± 0.15 | 1.93 ± 0.16* |  | 2.19 ± 0.12 | 1.63 ± 0.07** |  | <0.001 | 0.024 | n.s. |
|  | *grp-170* | 0.91 ± 0.06 | 0.92 ± 0.04 |  | 0.90 ± 0.05 | 0.77 ± 0.03* |  | n.s. | n.s. | n.s. |
|  | *grp-94* | 0.68 ± 0.03 | 0.73 ± 0.06 |  | 0.65 ± 0.05 | 0.68 ± 0.04 |  | n.s. | n.s. | n.s. |
|  | *mthsp70/grp-75/mortalin* | 1.58 ± 0.08 | 1.58 ± 0.13 |  | 1.52 ± 0.08 | 1.20 ± 0.03** |  | n.s. | 0.021 | n.s. |
| Xenobiotic metabolism | *ahr1* | 0.14 ± 0.01 | 0.14 ± 0.01 |  | 0.14 ± 0.01 | 0.12 ± 0.01 |  | n.s. | n.s. | n.s. |
|  | *cyp1a1* | 2.58 ± 0.25 | 3.63 ± 0.32* |  | 2.71 ± 0.23 | 3.79 ± 0.32* |  | <0.001 | n.s. | n.s. |
| Nuclear receptors | *gcr* | 1.01 ± 0.05 | 0.99 ± 0.04 |  | 1.05 ± 0.05 | 0.98 ± 0.09 |  | n.s. | n.s. | n.s. |
|  | *erα* | 0.17 ± 0.02 | 0.15 ± 0.01 |  | 0.19 ± 0.02 | 0.16 ± 0.01 |  | n.s. | n.s. | n.s. |
