# Supplementary material for: Tissue-Specific Orchestration of Gilthead Sea Bream Resilience to Hypoxia and High Stocking Density
Source: Front Physiol. 2019 Jul 10;10:840. doi: 10.3389/fphys.2019.00840 (PMC6635561; doi:10.3389/fphys.2019.00840)
Supplement: Supplementary file 7 [file Table_5.docx]

**Suppl. Table 5.** Effects of rearing density and dissolved oxygen level on gilthead sea bream relative expression of total blood cells genes on a 3-week feeding trial. Values on relative expression are the mean ± SEM of 8 fish (2-3 fish per replicate tank). P-values are the result of two-way analysis of variance. Non-significance (P>0.05) is stated by “n.s”. Asterisks in each row indicate significant differences with oxygen level for a given rearing density (SNK test, P<0.05).

| Category | Symbol | LD | |  | HD | |  | P-value | | |
| --- | --- | --- | --- | --- | --- | --- | --- | --- | --- | --- |
|  |  | Normoxia | Hypoxia |  | Normoxia | Hypoxia |  | [O_2_] | Density | Interaction |
| Antioxidant enzymes | *gst3* | 0.97 ± 0.07 | 1.09 ± 0.08 |  | 1.03 ± 0.07 | 0.90 ± 0.11 |  | n.s. | n.s. | n.s. |
|  | *prdx3* | 0.07 ± 0.01 | 0.08 ± 0.01 |  | 0.08 ± 0.01 | 0.07 ± 0.01 |  | n.s. | n.s. | n.s. |
|  | *prdx5* | 0.25 ± 0.02 | 0.23 ± 0.01 |  | 0.21 ± 0.03 | 0.21 ± 0.02 |  | n.s. | n.s. | n.s. |
|  | *Mn-sod / sod2* | 1.33 ± 0.09 | 1.36 ± 0.07 |  | 1.32 ± 0.06 | 1.22 ± 0.12 |  | n.s. | n.s. | n.s. |
| Transcription factors | *nrf1* | 0.95 ± 0.08 | 1.00 ± 0.09 |  | 1.01 ± 0.06 | 0.95 ± 0.08 |  | n.s. | n.s. | n.s. |
|  | *pgc1ß* | 1.71 ± 0.14 | 1.93 ± 0.13 |  | 1.95 ± 0.10 | 1.80 ± 0.11 |  | n.s. | n.s. | n.s. |
| Outer membrane translocation (*TOM complex*) | *tom70* | 1.10 ± 0.08 | 1.28 ± 0.08 |  | 1.20 ± 0.07 | 1.14 ± 0.09 |  | n.s. | n.s. | n.s. |
|  | *tom34* | 1.06 ± 0.08 | 1.16 ± 0.08 |  | 1.15 ± 0.07 | 1.05 ± 0.08 |  | n.s. | n.s. | n.s. |
|  | *tom22* | 0.57 ± 0.05 | 0.65 ± 0.05 |  | 0.62 ± 0.05 | 0.59 ± 0.05 |  | n.s. | n.s. | n.s. |
| Inner membrane translocation  (*TIM complex*) | *tim44* | 0.95 ± 0.08 | 1.12 ± 0.08 |  | 1.01 ± 0.06 | 0.96 ± 0.09 |  | n.s. | n.s. | n.s. |
|  | *tim23* | 0.10 ± 0.01 | 0.12 ± 0.01 |  | 0.12 ± 0.01 | 0.10 ± 0.01 |  | n.s. | n.s. | n.s. |
|  | *tim8a* | 1.02 ± 0.06 | 1.11 ± 0.06 |  | 1.08 ± 0.06 | 1.01 ± 0.07 |  | n.s. | n.s. | n.s. |
|  | *tim10* | 0.98 ± 0.08 | 1.13 ± 0.09 |  | 1.08 ± 0.07 | 0.99 ± 0.10 |  | n.s. | n.s. | n.s. |
|  | *tim9* | 1.36 ± 0.12 | 1.18 ± 0.08 |  | 1.14 ± 0.06 | 1.17 ± 0.09 |  | n.s. | n.s. | n.s. |
| Mitochondrial dynamics and apoptosis | *mfn2* | 0.45 ± 0.03 | 0.50 ± 0.04 |  | 0.50 ± 0.03 | 0.44 ± 0.04 |  | n.s. | n.s. | n.s. |
|  | *miffb* | 1.10 ± 0.09 | 1.23 ± 0.08 |  | 1.22 ± 0.07 | 1.14 ± 0.09 |  | n.s. | n.s. | n.s. |
|  | *miro1a* | 0.87 ± 0.08 | 0.86 ± 0.05 |  | 0.92 ± 0.06 | 0.94 ± 0.07 |  | n.s. | n.s. | n.s. |
|  | *miro2* | 1.17 ± 0.09 | 1.31 ± 0.08 |  | 1.38 ± 0.09 | 1.18 ± 0.07 |  | n.s. | n.s. | n.s. |
|  | *aifm1* | 0.07 ± 0.00 | 0.07 ± 0.00 |  | 0.08 ± 0.01 | 0.06 ± 0.00 |  | n.s. | n.s. | n.s. |
| FA oxidation & TCA | *cpt1a* | 1.64 ± 0.14 | 1.93 ± 0.16 |  | 1.75 ± 0.08 | 1.67 ± 0.13 |  | n.s. | n.s. | n.s. |
|  | *cs* | 1.10 ± 0.06 | 1.17 ± 0.08 |  | 1.13 ± 0.05 | 1.05 ± 0.06 |  | n.s. | n.s. | n.s. |
| OXPHOS (*Complex I*) | *nd2* | 10.19 ± 0.90 | 11.80 ± 0.82 |  | 11.12 ± 0.85 | 10.72 ± 0.77 |  | n.s. | n.s. | n.s. |
|  | *nd5* | 2.25 ± 0.18 | 2.68 ± 0.23 |  | 2.80 ± 0.27 | 2.55 ± 0.28 |  | n.s. | n.s. | n.s. |
|  | *ndufa1* | 1.03 ± 0.06 | 1.17 ± 0.08 |  | 1.08 ± 0.06 | 1.00 ± 0.08 |  | n.s. | n.s. | n.s. |
|  | *ndufa3* | 0.94 ± 0.05 | 0.93 ± 0.04 |  | 0.94 ± 0.06 | 0.85 ± 0.05 |  | n.s. | n.s. | n.s. |
|  | *ndufa4* | 1.84 ± 0.10 | 1.94 ± 0.06 |  | 1.97 ± 0.07 | 1.84 ± 0.07 |  | n.s. | n.s. | n.s. |
|  | *ndufa7* | 0.65 ± 0.05 | 0.69 ± 0.05 |  | 0.69 ± 0.04 | 0.66 ± 0.06 |  | n.s. | n.s. | n.s. |
|  | *ndufb5* | 0.88 ± 0.06 | 1.00 ± 0.07 |  | 0.91 ± 0.05 | 0.82 ± 0.07 |  | n.s. | n.s. | n.s. |
|  | *ndufs2* | 1.12 ± 0.07 | 1.24 ± 0.06 |  | 1.23 ± 0.07 | 1.13 ± 0.08 |  | n.s. | n.s. | n.s. |
|  | *ndufs7* | 0.75 ± 0.04 | 0.84 ± 0.05 |  | 0.83 ± 0.04 | 0.76 ± 0.05 |  | n.s. | n.s. | n.s. |
|  | *ndufaf2* | 0.11 ± 0.01 | 0.11 ± 0.01 |  | 0.11 ± 0.01 | 0.07 ± 0.01** |  | 0.034 | n.s. | 0.024 |
| OXPHOS (*Complex IV*) | *coxi* | 2.46 ± 0.22 | 2.95 ± 0.27 |  | 2.94 ± 0.27 | 2.36 ± 0.26 |  | n.s. | n.s. | 0.045 |
|  | *coxii* | 1.46 ± 0.12 | 1.91 ± 0.11* |  | 1.69 ± 0.14 | 1.46 ± 0.13 |  | n.s. | n.s. | 0.012 |
|  | *coxiii* | 3.21 ± 0.23 | 3.46 ± 0.32 |  | 3.89 ± 0.34 | 3.32 ± 0.44 |  | n.s. | n.s. | n.s. |
|  | *cox5a2* | 0.46 ± 0.05 | 0.45 ± 0.02 |  | 0.43 ± 0.02 | 0.46 ± 0.02 |  | n.s. | n.s. | n.s. |
|  | *cox6a2* | 0.25 ± 0.02 | 0.29 ± 0.02 |  | 0.28 ± 0.02 | 0.23 ± 0.01* |  | n.s. | n.s. | 0.047 |
|  | *cox6c1* | 0.20 ± 0.02 | 0.23 ± 0.03 |  | 0.22 ± 0.02 | 0.21 ± 0.03 |  | n.s. | n.s. | n.s. |
|  | *cox7b* | 0.73 ± 0.05 | 0.71 ± 0.04 |  | 0.83 ± 0.06 | 0.73 ± 0.06 |  | n.s. | n.s. | n.s. |
|  | *cox8b* | 1.14 ± 0.05 | 1.26 ± 0.07 |  | 1.19 ± 0.07 | 1.21 ± 0.10 |  | n.s. | n.s. | n.s. |
|  | *sco1* | 1.20 ± 0.10 | 1.36 ± 0.12 |  | 1.34 ± 0.12 | 1.22 ± 0.10 |  | n.s. | n.s. | n.s. |
|  | *surf1* | 0.76 ± 0.06 | 0.91 ± 0.08 |  | 0.90 ± 0.08 | 0.85 ± 0.08 |  | n.s. | n.s. | n.s. |
|  | *cox15* | 0.04 ± 0.00 | 0.05 ± 0.01 |  | 0.05 ± 0.01 | 0.04 ± 0.00 |  | n.s. | n.s. | n.s. |
| Respiration uncoupling | *ucp2* | 0.92 ± 0.10 | 0.99 ± 0.06 |  | 0.98 ± 0.06 | 0.88 ± 0.09 |  | n.s. | n.s. | n.s. |
